# Mitochondria Meet the Lung Microbiome: A Bidirectional Dialogue in Inflammation and Respiratory Diseases

**DOI:** 10.3390/biomedicines14091965

**Published:** 2026-08-31

**Authors:** Carola Parolin, Emanuele Gentile, Cristina Pellegrino, Valentina Spada, Cristian Bassi, Silvia Sabbioni, Beatrice Vitali, Paolo Pinton, Alessandro Rimessi

**Affiliations:** 1 Department of Pharmacy and Biotechnology, University of Bologna, 40126 Bologna, Italy; carola.parolin@unibo.it (C.P.); b.vitali@unibo.it (B.V.); 2 Department of Medical Sciences, Section of Experimental Medicine, and Laboratory for Technologies of Advanced Therapies (LTTA), University of Ferrara, Technopole of Ferrara, 44121 Ferrara, Italy; emanuele.gentile@unife.it (E.G.); cristina.pellegrino@unife.it (C.P.); valentina.spada@edu.unife.it (V.S.); 3 Department of Translational Medicine, Laboratory for Technologies of Advanced Therapies (LTTA), University of Ferrara, 44121 Ferrara, Italy; bsscst@unife.it (C.B.); silvia.sabbioni@unife.it (S.S.); 4 IRCCS Maria Cecilia Hospital, GVM Care & Research, 48033 Cotignola, Italy; 5 Center of Research for Innovative Therapies in Cystic Fibrosis, University of Ferrara, 44121 Ferrara, Italy

**Keywords:** mitochondria, microbiome, respiratory diseases, inflammation, dysbiosis

## Abstract

The respiratory tract is a dynamic biological interface where microbiome, environmental exposure, epithelial integrity, and host metabolic regulation converge to maintain pulmonary homeostasis. Once considered sterile, the lung is now recognized as a low-biomass yet structured microbial ecosystem that contributes to immune calibration, colonization resistance, epithelial barrier function, and tissue resilience. Disruption of this equilibrium, known as pulmonary dysbiosis, has been increasingly associated with acute and chronic lung diseases, including cystic fibrosis, chronic obstructive pulmonary disease, acute respiratory distress syndrome, idiopathic pulmonary fibrosis, asthma, bronchiectasis, and lung cancer. In parallel, mitochondria have emerged as central regulators of pulmonary cell function, extending beyond ATP production to control redox signaling, apoptosis, innate immunity, epithelial repair, and inflammatory responses. This review examines the bidirectional crosstalk between the respiratory microbiome and mitochondria as an integrated pathogenic axis in lung disease. Dysbiotic microbial communities and respiratory pathogens can induce mitochondrial stress through toxins, virulence factors, microbial metabolites, and pattern-recognition receptor activation, leading to mitochondrial alteration and the release of mitochondrial damage-associated molecular patterns. Conversely, dysfunctional mitochondria reshape the pulmonary microenvironment by altering oxygen consumption, nutrient availability, cytokine production, redox balance, and barrier repair, thereby favoring pathogen persistence and chronic inflammation. Understanding mitochondria–microbiome interactions may support precision medicine strategies that integrate microbial, metabolic, inflammatory, and bioenergetic biomarkers to improve the diagnosis, prognosis, and treatment of inflammatory-related lung diseases.

## 1. Introduction

The respiratory tract represents a complex biological interface in which environmental exposure, microbial colonization, and host metabolic regulation converge to sustain tissue homeostasis. Historically regarded as sterile, the lungs are now recognized as harboring structured and dynamic microbial communities whose composition varies across anatomical niches and disease profiles, thereby influencing immune tone and clinical outcomes [1].

Accumulating evidence further indicates that alterations in respiratory microbiome are associated with chronic pulmonary disorders, suggesting the centrality of host–microbe ecological balance in both physiological maintenance and pathological progression [2]. This evolving body of work has driven a conceptual transition in respiratory biology, reframing the microbiome as a potential contributor of disease processes rather than a passive correlate of pathology.

To date, mitochondria have gained recognition as pivotal regulators of pulmonary cellular function beyond their canonical ATP production. Mitochondrial dynamics, biogenesis, and mitochondrial stress responses govern epithelial barrier integrity, apoptotic susceptibility, and immune activation, collectively shaping tissue resilience and inflammatory responsiveness [3].

Experimental findings additionally indicate that mitochondrial responses influence airway epithelial barrier properties through integrated metabolic and redox-sensitive pathways, further linking mitochondrial physiology to structural and immunological homeostasis within the lung. About this, mitochondria may be considered as determinants to define the pulmonary microenvironments, thereby influencing microbial colonization patterns and persistence.

The existence of a bidirectional interactions between mitochondria and respiratory microbiome could catalyze increasing interest. In fact, experimental studies in other mucosal systems have demonstrated that host mitochondrial dysfunction reshapes microbial composition and inflammatory responses, suggesting the broader principle that bioenergetic disturbances can remodel ecological niches modifying microbial populations [4]. This conceptual framework could be progressively extended to respiratory biology, where mitochondrial activities may influence microbial community architecture, controlling nutrient flux, oxidative stress, and immune mediator release, while microbial-derived signals and metabolites reciprocally modulate mitochondrial homeostasis in epithelial and immune cells. Such mitochondria–microbiome crosstalk could emerge as a potential contributor of tissue-level homeostasis.

The clinical implications of this crosstalk are increasingly evident in inflammatory-related pulmonary disorders. Respiratory dysbiosis has been associated with immune dysregulation and disease progression in conditions including interstitial lung disease, asthma, cystic fibrosis (CF), and chronic obstructive pulmonary disease (COPD), suggesting that microbial composition reflects and potentially influences inflammatory responses and clinical outcomes [5,6,7,8,9]. In parallel, mitochondrial dysfunction contributes to epithelial injury, heightened immune activation, and impaired resolution of inflammation across diverse pulmonary pathologies [10,11,12,13]. Considered together, these observations raise the possibility that mitochondria-driven alterations in the pulmonary microenvironment foster microbial shifts that amplify inflammatory signaling, while reciprocal microbial perturbations further modulate host bioenergetics, establishing self-reinforcing pathogenic feedback loops.

Collectively, these observations delineate an expanding interdisciplinary field at the convergence of bioenergetics, microbial ecology, and inflammatory signaling. A comprehensive understanding of how mitochondrial regulatory processes can shape the pulmonary niche, and how microbial communities reciprocally influence epithelial and immune cell and mitochondrial function, is essential for interpreting lung disease pathogenesis within an integrated systems-level framework. Accordingly, this review aims to critically examine intracellular mechanisms, inflammatory consequences, and possible translational implications associated with this potential host–microbe crosstalk.

## 2. Composition and Functions of the Pulmonary Microbiome in Healthy Status

**Figure 1:**
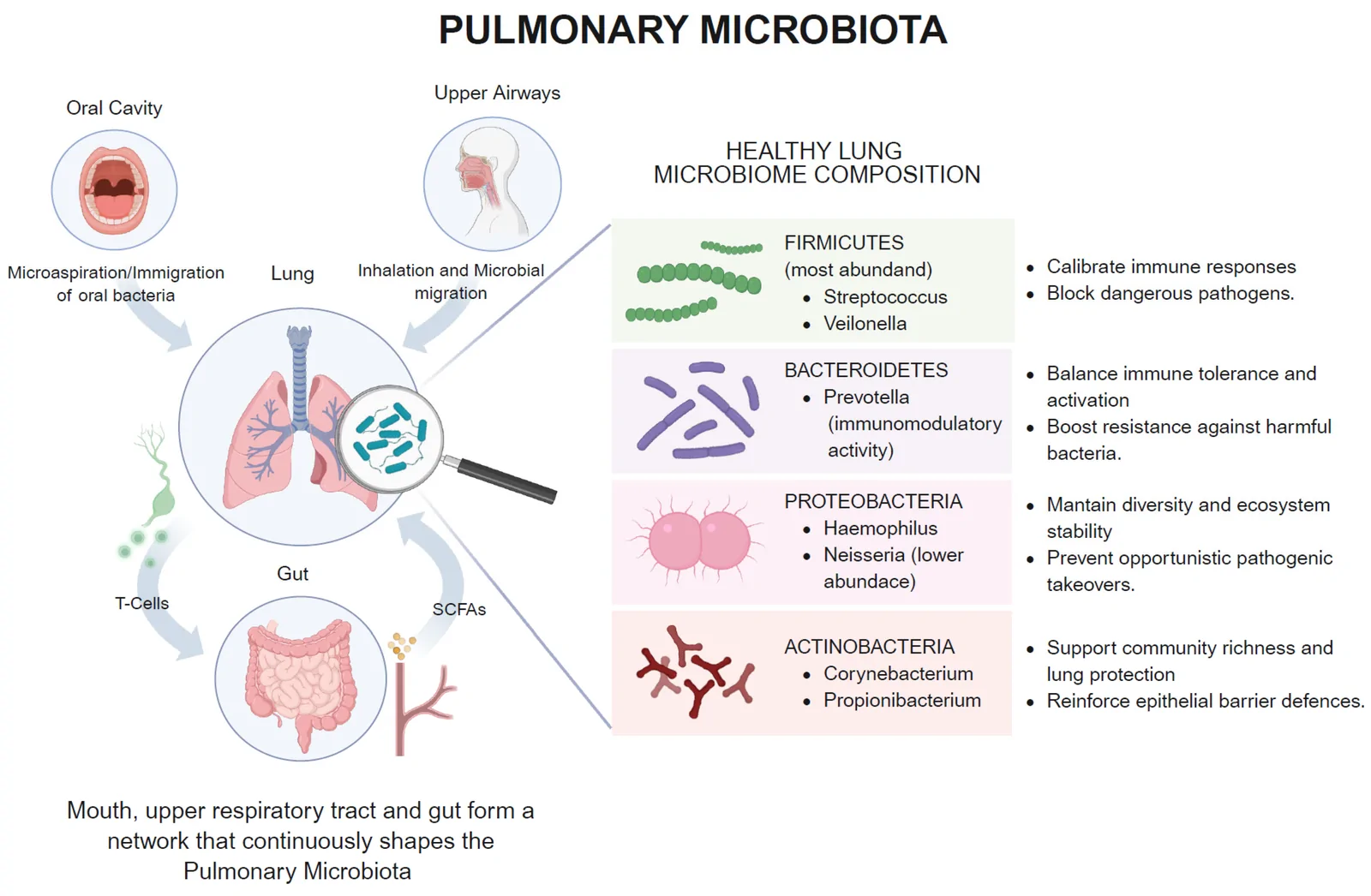
Composition and dynamics of representative bacteria commonly reported in the healthy pulmonary microbiome.

For much of the twentieth century, the lung was widely regarded as a sterile environment, presumed to be protected from microbial colonization by mechanical clearance mechanisms and host immune defenses [14]. This paradigm was fundamentally challenged with the introduction of culture-independent molecular methodologies, most notably 16S rRNA gene sequencing, which demonstrated that healthy lungs harbor diverse microbial communities [15]. These findings have substantially reshaped contemporary perspectives on pulmonary physiology, positioning the lung microbiome as an integral contributor to immune regulation and tissue homeostasis rather than a passive bystander [2]. Because the healthy lung represents a low-biomass microbial environment, characterization of its microbiome remains technically challenging. Reported community composition is influenced by several methodological factors, including specimen type, anatomical sampling site, oral contamination during bronchoscopy, DNA extraction procedures, sequencing platforms, and contaminant identification strategies [16]. Consequently, no universally accepted “core” healthy pulmonary microbiome has yet been established, although certain bacterial taxa are consistently reported across independent studies. Despite these technical challenges, studies consistently show that the lower respiratory tract harbors microbial communities with lower biomass (10^3^–10^5^ bacterial genomes per gram of tissue), reduced diversity, and greater temporal variability compared to those of the upper airways. Nevertheless, consistent representation of specific bacterial phyla suggests the existence of a structured, albeit dynamic, community rather than stochastic or purely transient colonization (Figure 1). Several studies have reported Firmicutes among the dominant phyla, with *Streptococcus* frequently identified as the most prevalent genus in healthy lungs [17]; however, relative abundance differs considerably between studies. The Firmicutes genus *Veillonella* is also commonly reported among the bacterial genera identified in healthy respiratory samples [18]. Members of the phylum Bacteroidetes, particularly *Prevotella*, are also commonly observed, although their reported abundance varies substantially among studies, and have been associated with immunomodulatory activity [19]. Proteobacteria, including *Haemophilus* and *Neisseria*, generally occur at lower abundance but remain characteristic components of the healthy pulmonary microbiome [20]. Actinobacteria, such as *Corynebacterium* and *Propionibacterium*, further contribute to overall community diversity [21].

The lung microbiome is continuously shaped by microbial immigration from the oral cavity (microaspiration) and upper airways (inhalation). It interacts with the gut via the gut–lung axis through T-cell migration and short-chain fatty acids (SCFAs) exchange. The right panel highlights the core phyla (*Firmicutes*, *Bacteroidetes*, *Proteobacteria*, *Actinobacteria*) and their essential roles in calibrating immune responses, maintaining ecosystem stability, and reinforcing epithelial barrier defenses.

Current ecological models indicate that the composition of the lung microbiome is determined primarily by the interplay of three processes: immigration from the upper airways, elimination, and local growth conditions [22,23]. Immigration occurs through microaspiration of oral secretions and inhalation of airborne particulates [24]. Elimination is mediated by mucociliary clearance, coughing, and innate immune activity [25]. Local microbial proliferation is shaped by nutrient availability and microenvironmental parameters, including oxygen tension and epithelial integrity within lung tissue [26]. This dynamic equilibrium accounts for the comparatively transient and variable nature of the lung microbiome relative to other microbial ecosystems, such as that of the gut, while preserving distinct functional characteristics [27].

Accumulating evidence indicates that the pulmonary microbiome fulfills multiple roles in maintaining respiratory health (Figure 1). Immune modulation represents a central function, as commensal microorganisms provide continuous low-level stimulation that calibrates host immune response [28]. For example, *Streptococcus* and *Prevotella* species have been implicated in shaping T-cell responses, thereby promoting equilibrium between tolerance and immune activation [29]. In addition, *Veillonella* spp. have been associated with the production of short-chain fatty acids capable of influencing both local and systemic immune processes [30]. Collectively, these observations highlight the microbiome’s role in immune education and regulatory homeostasis [31].

Colonization resistance has been proposed as a potential protective mechanism of the pulmonary microbiome. By occupying ecological niches and competing for resources, resident microbial communities may reduce opportunities for opportunistic pathogens to establish themselves [32]. While extensively documented in the gastrointestinal tract, evidence supporting a comparable role in the human lung remains limited and is derived largely from experimental models and indirect observations [33]. It has therefore been hypothesized that bacterial genera frequently detected in healthy lungs, including *Streptococcus*, *Veillonella*, and *Prevotella*, may contribute to limiting the expansion of potentially harmful organisms such as Pseudomonas aeruginosa (*P. aeruginosa*) or *Staphylococcus aureus*. However, the underlying mechanisms and their relevance in vivo require further investigation [34].

Beyond immune-related functions, microbial communities interact with the structural and biochemical environment of the lung. Certain bacteria produce metabolites that enhance epithelial barrier integrity, thereby increasing resistance to invasion [35]. Others modulate mucus secretion or influence ciliary function, indirectly reinforcing mechanical defense mechanisms [36]. Moreover, emerging evidence suggests that microbial signaling can regulate the expression of antimicrobial peptides, further strengthening tissue resilience [37].

The pulmonary microbiota should also be considered within the context of interconnected host-associated microbial ecosystems, particularly those of the oral cavity, upper airways, and gastrointestinal tract [38]. Nasal and nasopharyngeal microbiota act both as reservoirs and selective filters for microorganisms that may subsequently reach the lower respiratory tract, while the oral microbiota constitutes a major source of bacterial immigration to the lungs. Consequently, compositional shifts in upper airway communities may directly influence microbial migration patterns and the resulting pulmonary microbiome structure [39]. Additionally, increasing attention has been directed toward the gut–lung axis, which proposes bidirectional communication mediated by microbial metabolites and immune signaling pathways linking intestinal and pulmonary physiology [40]. Within this framework, the pulmonary microbiome forms part of a broader network of interacting microbial ecosystems collectively shaping host health [41].

In healthy individuals, this microbial equilibrium contributes to physiological resilience against disease. Disruption of community structure, commonly referred to as dysbiosis, has been associated with pathological conditions including asthma, COPD, and recurrent respiratory infections [42]. These states are frequently characterized by reduced microbial diversity or increased dominance of pathogenic taxa, which can promote exaggerated immune activation and chronic inflammation [43]. Conversely, maintenance of a balanced and diverse microbiome correlates with reduced susceptibility to such outcomes [28].

In summary, although no universally conserved bacterial composition has been defined for healthy lungs, numerous studies consistently report the detection of key bacterial genera, including *Streptococcus*, *Veillonella*, *Prevotella*, *Haemophilus*, *Neisseria*, *Corynebacterium*, and *Propionibacterium*. Their relative abundance, however, varies according to both biological and technical factors. These organisms contribute to immune modulation, colonization resistance, and preservation of tissue integrity. Their characterization provides important insight into pulmonary ecological dynamics, while their functional roles underscore the relevance of microbial communities to respiratory health. Continued investigation is likely to establish the pulmonary microbiome not only as a biomarker of physiological status but also as a potential therapeutic target, opening new avenues for prevention and management of respiratory disease [44].

### 2.1. Dysbiosis, Microbial-Derived Biomarkers and Molecular Signatures in Pulmonary Diseases

The lower respiratory tract harbors a dynamic pulmonary microbiome composed of bacteria, fungi, and viruses, which contributes to immune homeostasis and defense against infections [28]. In healthy lungs, this microbiota interfaces with both innate and adaptive immunity, influencing cytokine production, T-cell differentiation, and epithelial barrier integrity; perturbations in these interactions are increasingly implicated in pathological states. Dysbiosis, defined as qualitative and functional alterations in microbial communities, can disrupt this equilibrium and has been associated with chronic inflammation, immune dysregulation, and disease progression across multiple pulmonary conditions, including CF, COPD, acute respiratory distress syndrome (ARDS), idiopathic pulmonary fibrosis (IPF), and lung cancer [22].

### 2.2. Cystic Fibrosis

**Figure 2:**
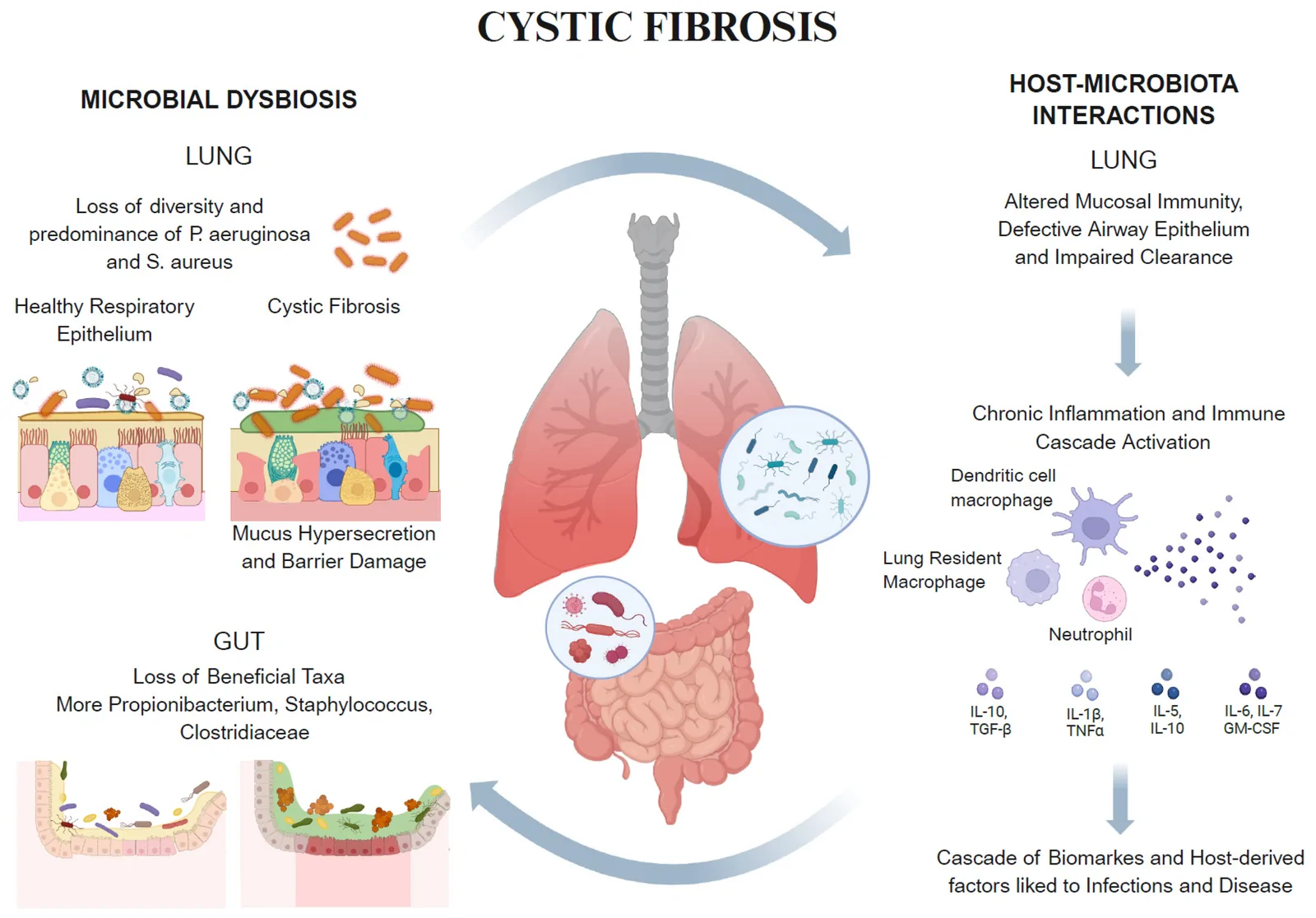
Microbial dysbiosis and host-microbiome interactions in cystic fibrosis.

CF is a genetic disease characterized from early life by marked pulmonary and intestinal dysbiosis that contributes substantially to disease manifestations [45]. In the airways, this imbalance is typified by a progressive loss of microbial diversity and the predominance of opportunistic pathogens, particularly *P. aeruginosa* and *Staphylococcus aureus*, which are associated with declining lung function and increased inflammation [33,46,47] (Figure 2).

In CF, *P. aeruginosa* infection progresses from early, potentially eradicable planktonic strains to chronic infection characterized by mucoid, biofilm-forming variants resistant to host defenses and antibiotics [48,49,50]. However, mucoid and non-mucoid phenotypes may coexist within different lung regions, generating spatially heterogeneous inflammatory responses [51,52,53]. This phenotypic plasticity may also contribute to pulmonary exacerbations through bacterial spread between differently colonized areas [54]. In addition to bacterial shifts, alterations in the airway mycobiome have been described; increased detection of fungi, such as Candida albicans, may further contribute to chronic airway inflammation [55]. However, these associations do not necessarily establish that dysbiosis is the primary cause of inflammation or disease progression, because antimicrobial exposure, structural lung damage, mucus obstruction, age, and disease severity may also shape airway microbial communities [56,57].

Disease progression triggers a severe loss of microbial diversity, marked by *P. aeruginosa* and *S. aureus* dominance in the lungs, accompanied by mucus hypersecretion, epithelial damage, and gut microbiome alterations. Impaired mucosal clearance and defective epithelium activate a chronic inflammatory cascade. This response is driven by macrophages, dendritic cells, and neutrophils, leading to a massive release of biomarkers and cytokines (including IL-1β, TNF-α, and IL-17) linked to infection.

In parallel, microbiome-informed biomarker research in CF has encompassed community-level signatures, microbial metabolites, and host-derived molecules influenced by microbial activity. Reduced microbial diversity, particularly a Shannon diversity index <1, has been proposed as a predictor of progression to early end-stage lung disease. Pseudomonas dominance, defined as ≥50% relative abundance, has been associated with poor outcomes, whereas *Streptococcus* abundance (≥25%) has shown potential protective associations [58,59] (Figure 2). However, these values should be interpreted as cohort-specific associations rather than universally applicable clinical thresholds, because relative abundance and diversity estimates are influenced by specimen type, sequencing depth, bioinformatic processing, antimicrobial treatment, and the characteristics of the investigated population [56,57].

Reported microbial metabolites include Pseudomonas-derived quorum-sensing signals quinolones, and phenazines such as pyocyanin have been detected in sputum from patients with bronchiectasis and CF. Pyocyanin has been detected at concentrations of up to approximately 16.5 and 27 µg/mL, respectively [60,61,62]. Pyocyanin is a membrane-permeable phenazine pigment and a major *P. aeruginosa* virulence factor that promotes bacterial persistence and chronic lung infection [63]. It undergoes intracellular redox cycling by accepting electrons from Nicotinamide Adenine Dinucleotide (NADH) and Nicotinamide Adenine Dinucleotide Phosphate (NADPH) and transferring them to molecular oxygen, thereby generating Reactive Oxygen Species (ROS) and Reactive Nitrogen Species (RNS) [60,64,65,66]. These reactive species cause mitochondrial dysfunction, ATP depletion, DNA and membrane damage, protein modification, and apoptosis [67]. In addition, oxidative stress and Protein Kinase C activation reduce ciliary beat frequency through ATP depletion, impair mucociliary clearance, and promote mucus accumulation, persistent infection, and chronic airway inflammation in CF and COPD [68,69,70]. However, several in vitro studies have used concentrations exceeding 100 µg/mL, raising concerns regarding the physiological and clinical relevance of these experimental conditions [71].

N-(3-oxododecanoyl)-L-homoserine lactone is a quorum-sensing molecule secreted by *P. aeruginosa* that induces mitochondrial calcium (Ca^2+^) overload, oxidative stress, and mitochondrial dysfunction, ultimately promoting apoptosis in human peripheral blood lymphocytes [72]. This effect may contribute to immune impairment during pathogen infection by reducing lymphocyte survival.

Additional metabolites associated with pulmonary exacerbations include lactate, putrescine, and pyruvate, which have been linked to anaerobic bacteria [73]. Host-derived biomarkers modulated by the microbiome include sphingolipids, bile acids, and amino acids. Sphingolipids have been reported among the most differentially abundant molecules in CF sputum and correlate with active neutrophil elastase and chronic pulmonary inflammation [74]. In particular, ceramides, sphingomyelins, and lactosylceramides have been identified through metabolomic profiling as dysregulated and differentially abundant molecules in CF biological samples. They contribute to chronic lung hyperinflammation, altered cellular signaling, and mitochondrial stress in CF airways [73]. Intracellular accumulation of ceramides directly affects mitochondrial membranes and mitochondrial network dynamics [75].

Bile acids detected in CF airway correlate with increased levels of pro-inflammatory mediators, including interleukin-1β (IL-1β) and IL-6. Aspirated or dysregulated bile acids may therefore link microaspiration and gut–lung metabolic alterations with structural lung disease and mitochondrial dysfunction [76,77]. Altered amino acid profiles have also been associated with inflammation and bacterial burden [73,76,78]. Free amino acids accumulate in lower-airway fluids (sputum and bronchoalveolar lavage fluid) from patients with CF, and provide a nutrient source that supports the growth and survival of pathogens such as *P. aeruginosa* [79]. The CF metabolome has been characterized by increased amino acid levels and reduced acylcarnitine levels. Both amino acids and acylcarnitines were, among the features, most strongly correlated with inflammation and bacterial burden. Random Forest analysis identified strong metabolomic predictors of CF status, including L-methionine-S-oxide. Spectrum-space collaborative network analysis also identified correlations between the metabolome and the microbiome, including associations involving the conventional CF pathogen, *Staphylococcus*, non-traditional taxa such as *Prevotella*, and a subnetwork of specific metabolomic markers [78].

Vernocchi et al. developed a predictive and functional model of the gut microbiome enterophenotype in clinically stable pediatric patients with CF, identifying a CF-specific microbial and metabolic profile. This enterophenotype was characterized by an increased abundance of *Propionibacterium*, *Staphylococcus*, and members of the family Clostridiaceae, together with depletion of beneficial taxa such as *Faecalibacterium prausnitzii* and Lachnospiraceae [80] (Figure 2). It was also associated with metabolic alterations, including increased levels of 4-aminobutyrate (GABA), choline, ethanol, and propylbutyrate, and decreased levels of sarcosine, glucose, and acetate [80]. These findings indicate that integration of microbial composition and metabolic profiling provides a more comprehensive representation of CF-associated intestinal dysfunction than culture-based methods alone [80].

In CF, both the gut and lung microbiomes are altered as a consequence of CF Transmembrane Conductance Regulator (CFTR) dysfunction, although local factors, including viscous mucus, chronic inflammation, and organ-specific environmental conditions, independently shape microbial communities at each site. Despite this partial independence, the two compartments are functionally connected through the gut–lung axis, primarily through the exchange of microbial metabolites [81]. In particular, the reduced abundance of short-chain fatty acid (SCFA)-producing bacteria in the CF gut may impair systemic and pulmonary immune regulation, contributing to defective lung immune homeostasis [82,83]. Experimental evidence further indicates that antibiotic-induced intestinal dysbiosis increases pulmonary hyper-reactivity in CF mouse models [84]. Therefore, repeated antibiotic exposure and the resulting loss of gut microbial diversity and metabolic potential may contribute to worsening pulmonary disease in patients with CF [85].

The respiratory epithelium represents the first chemical, immune, and physical barrier against inhaled harmful agents, particularly pathogens, in CF. In the CF bronchial epithelium, CFTR dysfunction causes mucus thickening, reduced airway-surface pH, and impaired mucociliary clearance, thereby promoting bacterial growth and chronic inflammation [86,87]. These alterations, which are primarily initiated by CFTR-dependent abnormalities, are accompanied by epithelial injury, loss or dysfunction of ciliated cells, goblet-cell hyperplasia, and progressive impairment of barrier integrity [88,89]. Once infection is established, microbial products, including lipopolysaccharides (LPS), SCFA and antimicrobial compounds, together with host inflammatory responses, further enhance mucus production and airway remodeling, including epithelial–mesenchymal transition [90,91,92]. Inflammation promotes persistent mucus secretion, whereas mucus accumulation facilitates bacterial trapping and amplifies epithelial inflammation. Sterile inflammation caused by mucus plugging and mediated through IL-1 signaling further reinforces this self-sustaining pathological cycle [93]. Within this amplification loop, mitochondria may function as intracellular sensors and effectors linking microbial exposure with epithelial and immune responses. Intrinsic CFTR-dependent mitochondrial abnormalities increase epithelial susceptibility to infection-associated stress, whereas microbial products subsequently intensify mitochondrial dysfunction and inflammation [94,95,96,97].

Mucosal immunity mediated by immunoglobulin A (IgA) is crucial for neutralizing inhaled pathogens such as *P. aeruginosa*. However, in CF, both epithelial transport through the polymeric immunoglobulin receptor (pIgR) and IgA production are altered [98]. Cellular stress and chronic infections modulate IgA and pIgR expression, highlighting a bidirectional interaction between the microbiome and the respiratory epithelium [99,100]. Mechanistic evidence also supports a role for microbiome-related modulation of pulmonary innate immunity in CF through Toll-Like Receptor 4 (TLR-4)-mediated pathways. Loss of CFTR function has been reported to reduce innate immune responses by decreasing TLR-4 expression in airway epithelial cells [55]. This alteration impairs cytokine responses to bacterial LPS, suggesting that it may contribute to chronic *P. aeruginosa* colonization in CF airways [101,102]. Hara Levy et al. demonstrated that the plasma of patients with CF was enriched in mediators including Transforming Growth Factor-beta (TGF-β), IL-1 Receptor Antagonist, Granulocyte-Macrophage Colony-stimulating Factor (GM-CSF), Macrophage inflammatory protein-1β (MIP-1β/CCL4), and IL-7. These mediators may promote inflammatory transcriptional programs, including interferon- and Nuclear Factor Kappa-light-chain-enhancer of activated B cells (NF-κB)-related pathways, and may contribute to a persistently activated but inefficient immune response [103]. However, this circulating cytokine profile represents an association with CF disease and does not demonstrate that airway dysbiosis directly caused the observed systemic immune alterations (Figure 2).

Imbalances among T helper 1 (Th1), Th2, and Th17 lymphocyte subsets, together with altered regulatory T-cell (Treg) activity, have also been reported in CF. These immune alterations correlate with changes in microbial composition, reduced microbial diversity, age, impaired lung function, and disease progression, collectively contributing to chronic airway inflammation [104]. Such relationships should be interpreted as interconnected associations rather than as evidence that microbial changes alone drive adaptive immune dysfunction.

Microbial antigens, including LPS and membrane proteins from *P. aeruginosa*, bacterial exotoxins, fungal antigens from *Aspergillus*, and microbial metabolites such as SCFAs and other biofilm-derived products, may influence B-cell maturation and antibody production, thereby modulating systemic adaptive immunity [105]. Overall, these imbalances exacerbate chronic infections, reduce the efficiency of pathogen clearance, and maintain the persistent inflammatory environment characteristic of CF lung disease [100,102,106].

Current evidence therefore supports a multifactorial model rather than a simple linear pathway. CFTR dysfunction initially alters mucus hydration, epithelial defense, and mitochondrial homeostasis. The resulting airway environment favors microbial persistence, while selected microorganisms and their products can subsequently aggravate mitochondrial dysfunction, oxidative stress, inflammation, mucus abnormalities, and tissue injury. These processes may form a bidirectional feed-forward loop, although the strength of the supporting evidence differs among its individual components.

### 2.3. Chronic Obstructive Pulmonary Disease

**Figure 3:**
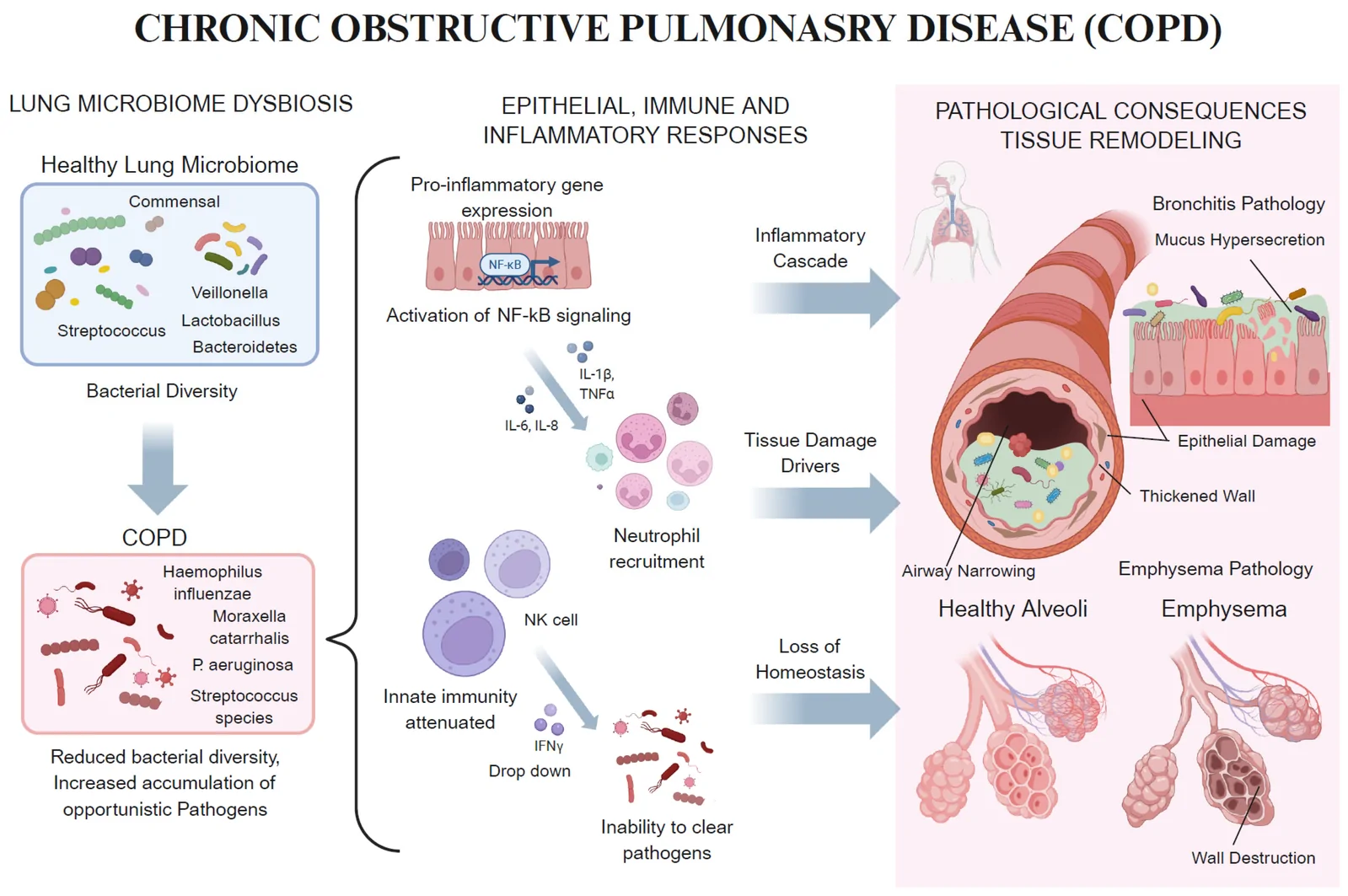
Lung dysbiosis, immune responses, and tissue remodeling in COPD.

Dysbiosis in COPD is characterized by alterations in airway microbial composition, with specific bacterial genera and molecular signatures strongly linked with disease progression and severity. Severe COPD has been reported to exhibit reduced microbial diversity and increased representation of potentially pathogenic bacteria, including Moraxella osloensis and *Streptococcus* species, which are associated with exacerbated neutrophilic activity [107,108] (Figure 3). This observation derives predominantly from cross-sectional or longitudinal association studies; they do not establish that the identified microbial changes directly cause COPD progression but define a specific scenario associated with pathology. These microbial patterns have also been linked to enhanced neutrophil extracellular trap (NET) formation, oxidative stress, and upregulation of NF-κB-dependent inflammatory programs, which contribute to airway remodeling and lung function decline. Host–microbe network analyses revealed strong correlations between these pathogens and disrupted immune-metabolic pathways, such as altered energy metabolism and inflammatory cascades [107].

Pulmonary dysbiosis reduces microbial diversity and increases opportunistic pathogens (*H. influenzae*, *M. catarrhalis*, *P. aeruginosa*) accumulation. This shift activates NF-kB signaling, upregulates pro-inflammatory cytokine expression, recruiting neutrophils and attenuating the protective role of innate immunity (NK cells and IFN-γ) against infection. These inflammatory drivers ultimately cause structural tissue remodeling, presenting as chronic bronchitis pathology (mucus hypersecretion and airway narrowing) and emphysema (alveolar wall destruction).

Dysbiosis of the lung microbiome and the consequent response of bronchial epithelial cells should play a key role in modulating inflammation and driving the progression of COPD (Figure 3). In patients with COPD treated with corticosteroids, *Prevotella* abundance was positively associated with expression of epithelial genes involved in tight junction promotion, while *Moraxella* abundance was associated with expression of IL-17 and TNF-α inflammatory pathways [108]. At the same time, inhaled irritants or cigarette smoke may also induce in bronchial epithelial cells a pro-inflammatory gene expression profile, characterized by upregulation of genes involved in innate immunity and NF-κB-mediated signaling, as well as the production of chemokines such as IL-8, chemokine C-X-C ligand 1 (CXCL1), CXCL5, CCL2 (MCP-1), and CCL20 [109,110]. These changes suggest that they may also be triggered independently of microbiome alterations, although the microbiome is considered one of the principal interacting determinants of airway inflammation. Immunologically, the lung microbiome is increasingly recognized as a regulator of host inflammatory patterns. Based on cytokine profiles, COPD can be classified into three inflammatory endotypes: neutrophilic, eosinophilic, and mixed [111]. The most common is the neutrophilic endotype, in which airway epithelial stimulation by opportunistic pathogens typically present in the COPD lung microbiome, such as *Haemophilus influenzae*, *Moraxella catarrhalis*, *Streptococcus* spp., and *P. aeruginosa*, induces the release of chemokines (e.g., IL-17, CXCL1, CXCL5, CXCL8). This response drives neutrophil recruitment, oxidative stress, mucus hypersecretion, airway remodeling, and emphysema, characterizing the chronic inflammation associated with the neutrophilic endotype [111] (Figure 3).

A direct mechanistic connection between microbial signals and mitochondrial dysfunction is biologically plausible but remains partially demonstrated in human COPD. Airway epithelial cells and macrophages from patients with COPD frequently exhibit altered mitochondrial respiration, increased mitochondrial ROS production, impaired mitochondrial quality control, and defective metabolic adaptation [10,112]. Microbial products such as LPS, bacterial proteases, and secreted toxins may aggravate these abnormalities by activating pattern-recognition receptors and redox-sensitive pathways. Conversely, mitochondrial dysfunction may amplify the epithelial response to microbial stimulation by increasing oxidative stress, facilitating NF-κB and inflammasome signaling, and reducing the energy available for epithelial repair, mucociliary transport, and macrophage phagocytosis [113]. Nevertheless, direct evidence that mitochondrial dysfunction itself modifies the composition of the COPD airway microbiome remains limited.

Beyond the airways, Bowerman et al. provided evidence that the gut microbiome and metabolome were altered in patients with stable COPD [114]. By integrating metagenomic and untargeted metabolomic approaches, the study identified a distinct COPD-associated gut microbial profile, characterized by enrichment of *Streptococcus* species and Lachnospiraceae members, and linked these changes to reduced lung function. The identification of disease-specific metabolic signatures, primarily involving lipid, xenobiotic, and amino acid metabolism, highlighted functional consequences of gut microbial dysbiosis. Integrative microbial–metabolic network analyses reinforced associations between *Streptococcus parasanguinis B* and COPD-related metabolites, such as N-acetylglutamate and N-carbamoylglutamate [114]. These findings support an association between gut microbial metabolism and COPD-related physiological alterations, but they do not establish that the identified microorganisms or metabolites directly drive pulmonary disease. A further hallmark described in COPD is the depletion of gut-derived SCFAs, particularly butyrate and propionate [115]. These changes may compromise intestinal barrier integrity, promoting the translocation of pro-inflammatory molecules, such LPS, into the systemic circulation [115]. Circulating inflammatory cues can subsequently impact the lung, amplifying airway inflammation and contributing to the systemic inflammatory state observed in COPD [115].

The interaction between the microbiome and mucosal immune cells induces metabolic and epigenetic modifications, which modify the immunological tolerance and attenuate pro-inflammatory responses [116,117,118]. Bacterial metabolites, such as butyrate and lactate, modulate the activity of transcription factors (e.g., NF-κB) and chromatin modifications, thereby regulating the expression of inflammatory and anti-inflammatory cytokines [119,120]. These mechanisms influence both innate immunity (dendritic cells, macrophages, neutrophils) and adaptive immunity, promoting the induction of regulatory T cells and inhibiting Th17 differentiation [121,122,123,124]. However, bacterial lactate may also act as a mitochondrial danger signal. In neutrophils exposed to *Staphylococcus aureus*, pathogen-derived lactate is transferred from bacteria-containing phagosomes to nearby mitochondria, where it promotes mitochondrial ROS production and contributes to NET formation [125].

Ganal et al. demonstrated that commensal microbiome provides essential instructive signals for the functional priming of natural killer (NK) cells. In germ-free or antibiotic-treated mice, NK cells showed impaired IFN-γ production and reduced antiviral responses. This defect was not due to a direct microbial effect on NK cells, but rather to insufficient activation of non-mucosal mononuclear phagocytes, which normally deliver cytokine signals, such as IL-12, required for NK cell maturation and activation [126]. These findings highlight a systemic role of the microbiome in shaping innate immunity.

### 2.4. Acute Respiratory Distress Syndrome

ARDS can be caused by various direct (e.g., aspiration) or indirect causes (e.g., sepsis) [127]; in any case, it is associated with significant dysbiosis of the lung microbiome, and this alteration appears closely linked to clinical course [128]. Patients with ARDS showed a significant decrease in diversity, and this index correlated with the length of stay in the intensive care unit and the need for mechanical ventilation [128]. Montassier et al. demonstrated that in patients with Acute Respiratory Failure (ARF) and Hospital-Acquired Pneumonia (HAP), a lower airway microbiome displays distinct signatures associated with disease severity [129]. More severe cases are characterized by reduced microbial diversity and dominance of nosocomial pathogens, such as *P. aeruginosa*, *Staphylococcus aureus*, Acinetobacter spp., Stenotrophomonas maltophilia, and members of the Enterobacteriaceae, which correlate with heightened inflammation and worse clinical outcomes. In contrast, a more diverse microbiome enriched with oral commensals (*Streptococcus*, *Prevotella*, and *Veillonella*) is associated with less severe clinical presentations [129]. The reported signatures provided prognostic information in the investigated cohort beyond conventional microbiological testing, supporting further evaluation of the airway microbiome as a potential biomarker of lung injury and infection.

Kyo et al. analyzed the lower respiratory tract microbiome in patients with ARDS and its relationship with inflammation and hospital mortality. Using 16S rRNA gene sequencing of bronchoalveolar lavage fluid (BALF), the authors observed a reduction in microbial diversity and an increased total bacterial load in patients compared to controls [130]. Specifically, the decrease in Betaproteobacteria and the increase in bacteria, such as *Staphylococcus*, *Streptococcus*, and *Enterobacteriaceae*, were associated with elevated IL-6 levels and higher hospital mortality [130]. Despite the correlations among bacterial burden, microbial composition, inflammation, and outcome, the authors do not demonstrate that the detected microorganisms directly caused systemic inflammation or mortality. These findings may be considered within the broader framework of biomarker development in ARDS.

Pulmonary dysbiosis has been proposed to influence epithelial barrier integrity and local immune responses, potentially affecting susceptibility to opportunistic pathogens such as *P. aeruginosa* [131]. Commensal microbes may contribute to the production of antimicrobial peptides, such as defensins (α- and β-defensins), cathelicidin LL-37, lysozymes, and lactoferrin, in the mucus and regulate the activation of macrophages and regulatory T cells; when their composition is altered, these defenses are reduced, promoting a persistent inflammatory environment [131]. The interaction between microbiome, epithelium, and immune system is therefore crucial: dysbiosis may facilitate the infection and amplify the inflammatory response, which can contribute to tissue damage and to the severity and progression of ARDS [131]. However, the extent to which specific commensal taxa directly induce these factors in human ARDS remains uncertain.

Mitochondrial dysfunction provides a potential mechanistic link between severe infection, epithelial injury, and dysregulated inflammation in ARDS. Similar to other respiratory diseases, the accumulation of dysfunctional mitochondria compromises the mitochondrial ATP production, which leads to epithelial and endothelial barrier disruption, alveolar edema, and impaired gas exchange [132,133,134]. Pathogen-associated signals, inflammatory cytokines, hypoxia, and oxidative stress can impair mitochondrial respiration, disrupt membrane potential, and alter mitochondrial dynamics in alveolar epithelial and endothelial cells. These abnormalities increase mitochondrial ROS production and reduce ATP availability, thereby promoting epithelial apoptosis or inflammatory cell death, weakening intercellular junctions, and compromising alveolar fluid clearance and alveolar–capillary barrier integrity [135,136]. Damaged mitochondria may also amplify innate immune activation through the release of mitochondrial damage-associated molecular patterns, including mitochondrial DNA, cardiolipin, and N-formyl peptides [137]. Human ARDS tissue has also been reported to exhibit mitochondrial oxidant damage together with defective mitochondrial quality-control responses in alveolar epithelial cells [134,138,139]. Nevertheless, few studies have simultaneously characterized the lung microbiome and mitochondrial function in the same patients. Therefore, the proposed microbiome–mitochondria interaction in ARDS currently rests on the integration of separate lines of evidence rather than on direct demonstration of a bidirectional pathway.

In the broader context of gut–lung immune interactions, fundamental mechanisms characterized in asthma and viral injury models provide essential insights into how the gut microbiota may modulate the severe systemic hyper-inflammation characteristic of ARDS. Trompette et al. showed that gut microbiota metabolism of dietary fiber generates circulating SCFAs that influence immune responses. SCFAs, particularly propionate, acted on the bone marrow to modulate hematopoiesis, enhancing the generation of macrophage and dendritic cells with reduced capacity to promote allergic Th2 responses [140]. Mice receiving a high-fiber diet exhibited increased circulating SCFAs and attenuated allergic airway inflammation, whereas a low-fiber diet worsened disease, supporting a systemic gut–lung immune axis mediated by microbial metabolites [140]. While originally described in allergic airway disease, this metabolite-driven bone marrow conditioning represents a crucial systemic axis; in the context of ARDS, an impairment of SCFA production, often caused by gut dysbiosis in critically ill patients, could similarly deplete tolerogenic immune populations and exacerbate acute alveolar damage. Beyond metabolite signaling, commensal bacterial signals are equally vital for maintaining basal pulmonary immune priming, a process that determines how the lung responds to acute inflammatory insults such as viral-induced ARDS. Ichinohe et al. highlighted that commensal microbiota, particularly antibiotic-sensitive gut bacteria, produce natural ligands, such as LPS and peptidoglycans, that activate TLRs to maintain immune readiness against respiratory pathogens like influenza [141]. This microbiota-driven signal supports the basal expression of pro-IL-1β and pro-IL18, facilitating inflammasome activation, dendritic cell migration, and an optimal T-cell responses. When this commensal crosstalk is disrupted, (e.g., through broad-spectrum antibiotic use in intensive care unit patients), the loss of basal TLR stimulation and inflammasome priming can severely compromise the host’s ability to control secondary lung infections or resolve hyper-inflammatory responses.

The gut–lung axis represents a bidirectional communication network through which intestinal microbial communities and ARDS may reciprocally influence one another. ARDS and the associated systemic inflammatory response can disrupt intestinal microbial homeostasis, impair epithelial tight-junction integrity, and increase gut permeability, thereby facilitating the translocation of microorganisms, microbial products, and inflammatory mediators into the systemic circulation [142,143]. Conversely, gut dysbiosis and the loss of microbiota-derived protective metabolites may contribute to the onset and progression of ARDS by altering innate and adaptive immune responses, promoting systemic and pulmonary inflammation [144,145]. Thus, gut and lung dysfunction may establish a self-amplifying cycle in which intestinal barrier damage, microbial imbalance, and pulmonary inflammation mutually reinforce disease severity.

### 2.5. Idiopathic Pulmonary Fibrosis

Alterations in the respiratory microbiome are also linked to disease progression and clinical outcomes in idiopathic pulmonary fibrosis (IPF). Integrated host-transcriptomic and microbial analyses have identified increased expression of host-defense genes, such as Secretory Leukocyte Protease Inhibitor (SLPI) and cathelicidin Antimicrobial Peptide (CAMP), in association with increased bacterial burden and enrichment of specific taxa, including *Neisseria*, *Streptococcus*, and *Staphylococcus* [83,84]. These features have been associated with inflammation, neutrophilia, disease progression and reduced survival.

Microbial communities appear to modulate innate immune signaling through TLR, NOD-like receptor (NLR), and RIG-I pathways, including NLR family CARD Domain Containing 4 (NLRC4), Peptidoglycan Recognition Protein 1 (PGLYRP1), Matrix metalloproteinase-9 (MMP9), and Defensin Alpha 4 (DEFA4), thereby influencing fibroblast activation and shaping Th1-polarized immune responses [146]. Huang et al. further reported that suppression of these key immune and inflammatory pathways, the presence of specific operational taxonomic units, TLR9 activation, and the accumulation of CXCR3^+^ CD8^+^ T cells were associated with accelerated disease progression [146]. These studies primarily demonstrate associations between microbial features and host-response patterns. They do not establish that microbiome alterations are the initiating cause of fibrosis or that the identified taxa directly activate all the reported immune pathways. A more cautious interpretation is that an altered or enriched lower-airway microbiome may provide persistent microbial stimulation in a subset of patients, potentially aggravating inflammation or repetitive epithelial injury in a susceptible host in IPF.

Metagenomic studies further indicated that the lung microbiome in IPF is characterized by reduced β-diversity and a distinct dysbiotic signature. IPF patients exhibit enrichment of *Streptococcus*, *Pseudobutyrivibrio*, and *Anaerorhabdus*, with a substantial proportion of bacteria originating from the oral cavity, consistent with microbial translocation to the lower airways [147]. Functional analyses showed increased abundance of microbial genes involved in ABC transporter systems, biofilm formation, and two-component regulatory pathways. Mechanisms composed by a sensor kinase and a response regulator that allow bacteria to sense and respond to environmental changes by modulating gene expression, indicating a broad adaptive capacity of bacteria within the lung microbiome of IPF patients [147]. Alterations in the pulmonary microbial community have been proposed to affect local immune regulation local immune regulation through TLR- and NLR-dependent pathways, NF-κB and MAPK signaling, and inflammasome activation [148]. These pathways can promote the production of TNF-α, IL-1β, IL-6, IL-17, TGF-β, CCL2, and CXCL8 and may influence macrophage activation, T-cell polarization, fibroblast responses, and extracellular-matrix deposition [148]. Nevertheless, most human microbiome studies in IPF are observational and cannot distinguish whether dysbiosis precedes fibrosis, develops as a consequence of architectural distortion and impaired clearance, or participates in a bidirectional interaction with tissue injury.

Mitochondrial dysfunction is independently well documented in alveolar epithelial cells, fibroblasts, and macrophages in experimental pulmonary fibrosis and IPF tissue [149]. Reported abnormalities include mitochondrial ROS accumulation, impaired mitophagy, altered mitochondrial biogenesis, metabolic reprogramming, and reduced stress tolerance [133,150,151]. These defects can promote epithelial senescence, profibrotic macrophage phenotypes, fibroblast activation, and resistance of myofibroblasts to apoptosis [152]. At present, however, direct studies connecting specific IPF-associated microbial communities to these mitochondrial abnormalities remain limited. Microbial ligands may plausibly aggravate mitochondrial stress through pattern-recognition receptor activation and inflammatory ROS production, whereas defective mitochondrial quality control may impair epithelial repair and antimicrobial defense. Whether these mitochondrial changes alter bacterial burden or community composition has not been directly established. Thus, to date, microbiome alterations and mitochondrial dysfunction should be presented as potentially convergent components of IPF pathogenesis rather than as a fully demonstrated causal feedback loop.

### 2.6. Lung Cancer

The human microbiome is increasingly investigated as a potential contributor to cancer development and progression [153]. Cheng et al. reported that the lung microbiome differed significantly between patients with lung cancer, enriched by *Saccharibacteria*, *Capnocytophaga*, *Sediminibacterium*, and *Gemmiger*, and individuals with benign pulmonary diseases, with clear separation of microbial communities in β-diversity despite no relevant differences in α-diversity [153]. Predictive functional analyses suggested enrichment of bacterial activity in metabolic pathways related to ribosomal biosynthesis and pyrimidine metabolism. When combined with clinical markers, selected microbial features showed potential diagnostic value [153]. Moreover, the diagnostic performance of these signatures requires validation in independent cohorts and careful control for smoking, medication, oral health, tumor location, bronchoscopy-associated contamination, and other potential confounders. The lower airway microbiome of patients with non-small cell lung cancer (NSCLC) is significantly enriched in bacterial taxa typically regarded as oral commensals, including *Veillonella*, *Prevotella*, and *Streptococcus*, compared with disease controls [154]. Notably, the presence of these microbial signatures was associated with the dysregulation of oncogenic signaling pathways implicated in lung carcinogenesis, particularly the PI3K pathway. Several other studies have identified tumor-associated microbial profiles, such as oral taxa *Megasphaera* and *Streptococcus*, in lung cancer patients compared with healthy controls or individuals with benign pulmonary diseases [155]. Additional microbial signatures, including Pseudomonadaceae, Capnocytophaga, Stenotrophomonas, and Microbacterium, have been reported to enhance diagnostic performance, particularly when combined with established clinical tumor markers, such as carcinoembryonic antigen (CEA), Cytokeratin 19 fragments (CYFRA21-1), and Neuron-specific Enolase (NSE) [155]. Moreover, enrichment of Clostridiales and Bacteroidales has been associated with post-surgical recurrence in early-stage NSCLC, outperforming conventional clinical predictors in some analyses. Despite these promising observations, most studies remain limited by small cohort sizes, lack of external validation, and clinical heterogeneity, emphasizing the need for standardized and prospective validation efforts [155]. Associations with diagnosis, recurrence, or signaling-pathway activation do not demonstrate that the identified bacteria initiate or promote cancer in humans.

From a mechanistic perspective, Invernizzi et al. reported that lung epithelial cells actively sense microbial signals through pattern recognition receptors, particularly TLRs, thereby orchestrating innate and adaptive immune responses [156]. Cheng et al. further indicated that respiratory symbionts may either promote or inhibit tumor progression through immune activation, modulation of DNA damage, and effects on genomic instability [157]. The direction and magnitude of these effects are likely to depend on microbial identity, anatomical compartment, host genotype, tumor stage, and immune context. Chow et al. demonstrated that Gram-negative bacteria promoted NSCLC metastasis by activating TLR4. TLR4 stimulation triggered downstream p38 MAPK and ERK1/2 phosphorylation, enhancing adhesive, migratory, and metastatic behaviors of lung cancer cells in vivo [158]. This study provides mechanistic evidence for a bacterial ligand-host signaling pathway but should not be generalized to the entire lung microbiome or all forms of NSCLC.

Consistent with a functional role for the microbiome, Hu et al. showed that manipulation of the microbiome led to a significant reduction in lung lesions and reshaped NK and CD8^+^ T-cell populations [159]. Microbiome perturbation changed IFN-γ production and antitumor immune surveillance, and the CXCL9–CXCR3 axis was implicated in the recruitment of NK and CD8^+^ T cells [159].

Mitochondria may represent an intersection between microbial signaling and tumor biology, but direct evidence remains very limited [160]. TLR activation and inflammatory cytokines can alter mitochondrial ROS production, oxidative phosphorylation, and metabolic substrate use in cancer and immune cells [161,162,163,164]. In parallel, tumor-cell mitochondrial metabolism influences proliferation, resistance to stress, metastatic competence, and the composition of the tumor immune microenvironment [165,166,167]. A potential model is that persistent microbial stimulation modifies mitochondrial and redox signaling in epithelial or tumor cells, thereby affecting oncogenic pathways and immune-cell function. Conversely, tumor-associated changes in oxygen consumption, nutrient availability, necrosis, mucus production, and local immunity may create ecological niches that select microbial communities. However, the latter direction represents an inference based on tumor physiology, and direct evidence that mitochondrial alterations in lung cancer cells determine airway or intratumoral microbiome composition is currently insufficient.

## 3. Microbial Dysbiosis, Mitochondrial Stress, and Mechanisms of Pulmonary Tissue Injury

Normal lung function relies on mitochondrial ATP production to sustain airway and smooth vascular muscle activity required for efficient gas exchange. Beyond bioenergetics, mitochondria regulate key protective processes, such redox homeostasis, calcium (Ca^2+^) signaling, innate immune response and programmed cell death. When oxygen tension declines, due to impaired ventilation or gas exchange, oxidative phosphorylation may become restricted and cells can increase their reliance on anaerobic glycolysis, leading to lactate accumulation and reduced mitochondrial ATP production [135,168].

Mitochondrial dysfunction in lung in lung epithelial and immune cells exposed to respiratory pathogens or microbial products has increasingly been recognized as an important component of inflammatory and infectious lung pathophysiology [169]. Available evidence supports pathways in which selected microorganisms or their virulence factors alter mitochondrial Ca^2+^ handling, membrane potential, respiration, metabolism, dynamics and redox signaling. Mitochondrial injury may be associated with oxidative damage, impaired organelle quality control mechanisms, and extracellular or cytosolic release of ROS, and of mitochondrial damage-associated molecular patterns (mtDAMPs), including mitochondrial DNA (mtDNA), cardiolipin, ATP and N-formyl peptides, and mitochondrial-derived vesicles as pro-inflammatory signaling, which potentially modify selected ecological pressures within the airway environment rather than directly determining the composition of the pulmonary microbiome [10,12]. In turn, these mitochondrial alterations can affect epithelial integrity, inflammatory signaling, immune-cell metabolism and antimicrobial responses [170]. By contrast, direct evidence that mitochondrial dysfunction alone modifies the overall architecture of human pulmonary microbiome remains limited.

Although CF airway infections are polymicrobial throughout life, clinical and research attention has largely focused on *S. aureus* and *P. aeruginosa*, which are among the principal microorganisms associated with progressive CF lung tissue damage [171]. Exposure to *P. aeruginosa* induces profound mitochondrial alterations in CF airway epithelial cells, including swelling, fragmentation, and cristae disorganization, ultimately leading to loss of mitochondrial membrane potential, increased mitochondrial ROS production, and activation of NLRP3 and NLRC4 inflammasome [97]. Bacterial flagellin can active TLR5-dependent signaling and promote mitochondrial Ca^2+^ uptake, through the mitochondrial Ca^2+^ uniporter (MCU), thereby contributing to NLRP3 inflammasome activation and caspase-1-dependent processing of pro-IL-1β and pro-IL-18 [97]. Studies employing non-motile or flagellin-deficient *P. aeruginosa* mutants confirmed that these mitochondrial defects were largely driven by bacterial flagellin. Type 3 Secretion System (T3SS) of *P. aeruginosa* also promotes mitochondrial damage coupled to the release of ROS and mtDNA, which activates NLRC4 inflammasome in airway cells, which is actively contained by mitophagy [172]. By contrast, the effector protein ExoS favors *P. aeruginosa* survival in these cells by inhibiting autophagy mediating inhibition of mTOR, caused by ExoS-mediated ADP ribosylation of RAS, and by the suppression of autophagic Vps34 kinase activity [173].

The pathogenicity of *P. aeruginosa* is dependent on quorum sensing, an inter-bacterial communication system able also to modulate host epithelial cell biology [174]. 3-oxo-C12-HSL induces mitochondrial fragmentation, mitochondrial bioenergetic alterations, and mtDNA oxidative injury, affecting the expression of PGC-1a and TFAM and mitochondrial biogenesis [174]. These findings provide a direct mechanistic example of a microbial factor acting through mitochondrial signaling to amplify airway inflammation.

Klebsiella pneumoniae, a common cause of pneumonia, can induce mitochondrial stress in bronchial epithelial cells and in infected murine lungs in vivo through its siderophore enterobactin, with subsequent activation of NLRP3 inflammasome [175]. In pneumococcal pneumonia, the bacterial toxin pneumolysin induces mitochondrial Ca^2+^ overload, opening of the mitochondrial permeability transition pore (mPTP), mitochondrial depolarization, and ATP depletion, thereby promoting the release of mtDNA-enriched Extracellular Vesicles (EVs) that can amplify inflammatory response in recipient cells [176,177]. When internalized by neutrophils, these EVs have also been reported to impair ROS generation and antimicrobial functions [178].

The process may begin when microbial exposure or dysbiosis increases mitochondrial ROS production and promotes mitochondrial injury in epithelial or immune cells. Increased mitochondrial ROS may mediate lipid peroxidation, mitochondrial membrane damage, and oxidation of mtDNA. Oxidated mtDNA and mitochondrial ROS-dependent signaling are principally associated with activation of NLRP3 inflammasome, but NLRP3 and NLRC4 should not be merged into a single mitochondrial activation pathway. NLRP3 responds to a broad range of cellular perturbations, including ionic imbalance, mitochondrial dysfunction, and cytosolic oxidized mtDNA, assembling with ASC and pro-caspase 1 to promote the release of mature pro-inflammatory cytokines, IL-1β and IL-18 [179,180]. In contrast, NLRC4 is activated through NAIP-mediated recognition of bacterial components, such flagellin and T3SS, which contribute to pathogen-induced mitochondrial stress that triggers the leakage of oxidized mtDNA acting as a critical binding partner to NLRC4 activation and downstream inflammation [11,172,181]. Therefore, although *P. aeruginosa* may engage both NLRP3 and NLRC4 inflammasome, their upstream activation mechanisms are distinct.

IL1β and IL-18 have been associated with fibrotic and inflammatory processes in interstitial lung diseases and acute lung injury, and correlations between IL-1β levels and microbial burden in bronchoalveolar samples have been reported [182,183]. Such clinical associations, however, do not establish that microbiome alterations or mitochondrial dysfunction directly caused the observed cytokine profile. Stronger evidence for causality derives from in vitro and in vivo studies in which mitochondrial Ca^2+^ uptake, mitochondrial ROS production, or inflammasome components were experimentally manipulated.

Mitochondrial ROS have been also linked to mitochondrial membrane permeabilization and cytochrome *c* release, alterations documented in airway epithelial cells during bacterial pneumonia and *P. aeruginosa*-induced injury [174,182,183,184]. While *P. aeruginosa* triggers mitochondrial dysfunction and apoptosis through quorum-sensing molecules like 3-oxo-C12-HSL, which induces mitochondrial ROS generation, mitochondrial membrane permeabilization, and cytochrome *c* in epithelial and immune cells [174,184,185]. The pathogen simultaneously drives a highly inflammatory form of lytic cell death. Whereas apoptosis represents an immunologically silent pathway, *P. aeruginosa* also promotes pyroptosis, a process that specifically requires inflammatory caspase activation and Gasdermin-dependent membrane pore formation [186]. Mechanistically, intracellular detection of *P. aeruginosa* pathogen-associated molecular patterns, such flagellin or its T3SS, leads to the assembly of inflammasomes and subsequent activation of caspase-1. Concurrently, internalized LPS triggers the non-canonical caspase-4/5 pathway [187]. Both cascades converge on the proteolytic cleavage of Gasdermin D, where the fragments oligomerize at the host plasma membrane forming lethal pores and the massive release of mature IL-1β and IL-18.

Mitochondrial injury can also influence host metabolic programming, thereby altering inflammatory tone and the availability of metabolites within the pulmonary microenvironment. In airway epithelial cells and macrophages, mitochondrial dysfunction and inflammatory activation have been associated with shifts in substrate utilization and the accumulation of metabolites such as succinate [188,189]. Elevated succinate levels have been observed in association with alveolar epithelial dysfunction in ARDS and may stabilize hypoxia-inducible factor-1α (HIF-1α) by inhibiting prolyl-hydroxylases, thereby promoting the transcription of genes involved in glycolytic reprogramming and cytokine production [188,190].

Airway epithelial cells undergoing inflammation shift from oxidative phosphorylation toward glycolysis, reducing cellular oxygen consumption. It has been proposed that, under selected conditions, reduced epithelial oxygen consumption could alter oxygen availability at the epithelial-luminal interface. However, this mechanism should not be presented as a universally established explanation for pathogen expansion. Oxygen conditions within diseased airways are spatially heterogeneous and are also determined by ventilation, perfusion, mucus thickness, diffusion distance, microbial respiration, neutrophil activity, and biofilm formation. In CF mucus plugs, for example, relatively oxygenated surfaces may coexist with hypoxic or anoxic microdomains [191]. This distinction also reconciles the apparent contradiction between reduced intracellular oxygen availability, which can impair oxidative phosphorylation in host cells, and the possibility that reduced epithelial oxygen consumption may locally influence oxygen concentrations in the airway lumen. Accordingly, the expansion of facultative anaerobes, including *P. aeruginosa*, *S. aureus*, and members of the Enterobacteriaceae family such as Escherichia coli and Klebsiella pneumoniae, cannot be attributed solely to increased luminal oxygen availability [24,191,192,193,194]. Rather, inflammation, mucus obstruction, altered epithelial metabolism, immune-cell activity, nutrient release, antimicrobial exposure, and biofilm formation collectively generate heterogenous ecological niches that may favor the persistence of selected microorganisms.

In parallel, microbial-derived metabolites can directly interfere with host bioenergetics. Hydrogen sulfide (H_2_S), produced by several oral- and respiratory-associated microorganisms, including Fusobacterium spp. and *P. aeruginosa*, may inhibit cytochrome *c* oxidase at sufficiently high local concentrations, thereby suppressing mitochondrial respiration [195]. At lower concentrations, however, H_2_S may exert reversible or signaling effects, and its biological activity its strongly dependent on concentration, oxygen tension, and cellular redox status. Similarly, nitric oxide generated by bacterial enzymes or by host inducible nitric oxide synthase during inflammation can competitively bind to cytochrome *c* oxidase and transiently impair oxidative phosphorylation [196]. Therefore, the effects of these metabolites should be interpreted as context-dependent rather than uniform across all pulmonary compartments.

In CF airway epithelia, the disruption of CFTR-phosphatase and tensin homolog (PTEN) axis, in which CFTR contributes to the membrane localization and function of PTEN, results in altered mitochondrial and immunometabolic responses during *P. aeruginosa* infection [197,198]. Defective CFTR-PTEN signaling promotes the accumulation and release of immunometabolites, including itaconate and succinate [197,198]. Experimental evidence indicates that *P. aeruginosa* can adapt to succinate-enriched conditions and preferentially use this metabolite to sustain bacterial growth, biofilm production, and persistence. This adaptation may also modify competitive interactions with other airway microorganisms, including *S. aureus*. This mechanism provides direct evidence that a host mitochondrial and immunometabolic abnormality may modify the nutritional environment encountered by a specific pathogen. However, it does not demonstrate that mitochondrial dysfunction globally determines the composition of the CF airway microbiome. The resulting metabolic milieu may therefore provide a selective advantage to *P. aeruginosa* persistence in CF lungs [197,198]. Collectively, these mechanisms support a feed-forward interaction in which microbial factors can impair mitochondrial function, while mitochondrial and immunometabolic abnormalities may modify selected ecological conditions encountered by airway microorganisms.

The release of mtDAMPs in the extracellular milieu represents another important downstream consequence of mitochondrial injury that can shape pulmonary inflammatory responses. Extracellular release of intact mitochondria or mitochondrial components and metabolites may influence immune responses and local microenvironmental conditions. Oxidized mtDNA and other mitochondrial constituents may be released freely or packaged within EVs from stressed airway epithelial cells, where they act as alarmin signal [12,199]. Mitochondrial extrusion or transfer has also been described in airway smooth muscle cells and alveolar epithelial cells under inflammatory or mechanical stress conditions [200,201]. Extracellular mitochondria or EVs released by stressed structural lung cells may be taken up by alveolar macrophages, neutrophils, and dendritic cells. In recipient cells, mtDNA may activate TLR9-dependent signaling, promoting NF-κB-driven cytokine release and neutrophil recruitment [132,202], while mitochondrial injury and redox signaling may enhance NET formation and tissue damage [203]. Cytosolic mitochondrial components may also contribute to inflammasome activation, promoting IL-1β and IL-18 secretion [204]. Nevertheless, mitochondrial transfer and EV signaling are not invariably pro-inflammatory. Their effects depend on the phenotype of the donor cell, the integrity of the transferred mitochondria, vesicle composition, and the metabolic state of the recipient cell. Thus, mitochondrial transfer may represent either a damage-associated inflammatory signal or a compensatory mechanism that restores recipient-cell bioenergetics. In ARDS, increased circulating cell-free mtDNA has been associated with disease development, severity, and mortality and may reflect the extent of tissue and mitochondrial injury [205,206]. These associations support the potential value of mtDNA as a biomarker but do not, by themselves, demonstrate that extracellular mtDNA is the initiating cause of ARDS.

The activation of resident macrophages and other innate immune cells can be attenuated by interventions targeting mitochondrial dysfunction in experimental models [182]. The limited efficacy of conventional antioxidant therapies in clinical trials has stimulated interest in mitochondria-based approaches aimed at restoring cellular bioenergetics and reducing chronic inflammation [207,208,209,210,211]. Mitochondria-targeted antioxidants, such as mitochondria-targeted vitamin E (MitoVit-E) and mitoquinone (Mito-Q), have shown beneficial effects in preclinical inflammatory-related diseases [212]. MitoVit-E reduced IL-1β production and improved mitochondrial and cardiac function in sepsis, whereas Mito-Q restored mitochondrial activity and reduced inflammation and IL-8 release in COPD-related models [213,214]. Mesenchymal stem cell-derived CD44^+^ EVs containing functional mitochondria enhanced macrophage phagocytosis and promoted an anti-inflammatory M2 polarization in experimental systems [215,216].

EVs may disseminate mitochondrial and inflammatory signals beyond the lung, contributing to endothelial activation and systemic inflammatory propagation. Systemic modulators, including the gut–lung axis, may also influence airway epithelial resilience and repair, particularly during ARDS. ARDS-associated disruption of the gut–lung axis can increase intestinal permeability, potentially enabling microbial products and, in selected experimental settings, viable bacteria to enter the circulation and amplify pulmonary inflammation [217]. Experimental evidence supports distal gut–lung communication: depletion of beneficial taxa, such as Akkermansia muciniphila has been associated with aggravated alveolar injury, whereas probiotic supplementation restored epithelial integrity in preclinical models [218,219]. These findings support biological plausibility but do not establish therapeutic efficacy in human ARDS. In preclinical models, microbiome-targeted interventions, including Lactobacillus rhamnosus GG and Bifidobacterium breve, have restored Treg/Th17 balance, reduced pulmonary inflammation, and improved epithelial barrier function [144,220,221]. SCFAs, such as butyrate, have also attenuated inflammation and promoted epithelial or mucus barrier repair through pathways involving GPR41 (FFAR3) and GPR43 (FFAR2) [222,223].

The depletion of intracellular ATP availability and persistent redox imbalance, secondary to mitochondrial dysfunction, may compromise epithelial restitution, tight junction integrity, ciliary activity, and mucociliary clearance, potentially favoring pathogen persistence [217]. Impaired epithelial repair and defective mucociliary transport facilitate persistent bacterial colonization and biofilm formation, which are characteristic of chronic airway diseases, such as CF and COPD [214,224]. Bacterial virulence factors can further impair host defense. For example, the *P. aeruginosa* protease LasB interferes with host-defense mechanisms and bacterial killing by CF macrophages, thereby exacerbating innate immune dysfunction [225].

Microbial exposure or lung microbiome dysbiosis may activate selective autophagy programs in airway epithelial cells and macrophages, including mitophagy, the selective autophagic degradation of damaged mitochondria, and xenophagy, the selective targeting of intracellular pathogens. These pathways may be protective by limiting mitochondrial ROS production, preventing mtDAMPs release, and promoting bacterial clearance. However, increased recruitment or expression of individual autophagy proteins does not necessarily indicate complete and effective autophagic flux. In experimental model of IPF, tissue-associated *Klebsiella quasipneumoniae* has been linked to irregular mitochondrial morphology and increased mitochondrial ROS production in macrophages, recruitment of PINK1/Parkin machinery, and activation of redox- and Transforming Growth Factor-β-dependent profibrotic signaling [183]. This evidence supports a potential causal role in the experimental model, although most human lung microbiome studies in IPF are observational and cannot determine whether microbial alterations precede fibrosis or develop as consequence of structural injury, altered immunity, aspiration, medication, or disease progression.

While initially protective, insufficient or dysregulated mitophagic flux may result in the accumulation of damaged mitochondria, sustained mtROS production, and release of mtDAMPs, thereby amplifying inflammation and fibrotic progression [183]. Defective mitophagy and xenophagy may also contribute to mitochondrial dysfunction and hyperinflammation in CF. CF airway cells display impaired selective autophagic responses, resulting in the accumulation of dysfunctional mitochondria and reduced bacterial clearance, which favored intracellular bacterial persistence and pyroptosis [97,226]. In CF macrophages, the persistence of Burkholderia cenocepacia has been linked to altered p62-dependent cargo recognition and impaired xenophagic responses in CFTR-mutant macrophages [227]. Restoration of selected autophagic responses and mitochondrial integrity during *P. aeruginosa* infection was achieved by silencing or pharmacologically inhibiting MCU, supporting a role for mitochondrial Ca^2+^ signaling in pathogen-induced autophagic alterations and mitochondrial dysfunction in CF airway cells [97,226].

Other respiratory pathogens can also engage xenophagy in pulmonary cells. *Streptococcus pneumoniae* induces ubiquitin-dependent bacterial targeting and recruitment of selective autophagy receptors, such as p62/SQSTM1 and NDP52, which connect ubiquitinated cargo to LC3-decorated phagophores [228]. Similarly, *P. aeruginosa* may active canonical autophagy in alveolar macrophages through the Beclin-1-ATG5-ATG7 axis, contributing to bacterial clearance [229]. Conversely, several bacteria have evolved mechanisms to subvert selective autophagy. In airway epithelial cells, the *P. aeruginosa* T3SS effector, ExoS, can impair autophagosome biogenesis by inhibiting the ATG14L-VPS34 complex, thereby limiting xenophagic clearance [173]. Legionella pneumophila can directly block autophagosome maturation through the effector RavZ, which irreversibly deconjugates LC3 from autophagic membranes [230]. Collectively, these findings highlight a dual role for pulmonary bacteria in modulating selective autophagy: certain pathogens activate mitophagy and xenophagy as part of host response, whereas others actively disrupt these pathways to promote persistence. The balance between effective selective autophagy and its microbial subversion may influence inflammatory amplification, tissue injury, and disease progression in chronic lung disorders. These studies provide direct evidence that microorganisms can alter mitochondrial quality control and autophagic pathways. Conversely, the extent to which altered mitophagy or mitochondrial function modifies the overall composition of the human lung microbiome remains limit established, resulting in an intriguing area for future investigations.

## 4. Methods to Determination, Clinical Relevance of the Pulmonary Microbiome and Therapeutic Perspectives

The determination of the pulmonary microbiome requires a particularly careful methodological approach, mainly because the lung is considered a low-biomass environment, shaped by continuous micro-aspiration, mucociliary clearance, and local immune responses [231,232]. Unlike the gut or the oral cavity, the lower airways contain relatively small amounts of microbial material, and this makes every phase of the analysis highly sensitive to contamination. For this reason, in the study of the pulmonary microbiome, the focus must be on the quality of sampling strategy to avoid contamination.

BALF and protected specimen brush (PSB) samples are commonly used methods to investigate the pulmonary microbiome, but they have distinct methodological limitations. BALF samples a relatively broad distal airway and alveolar compartment; however, it is not intrinsically protected from upper-airway carryover because the bronchoscope passes through the oral or nasal cavity and the pharynx. This limitation is particularly relevant in low-biomass samples, in which even minor contamination may substantially affect microbial profiles [15,233,234,235]. BALF is also influenced by saline dilution and variable fluid recovery. PSB reduces procedural carryover through its protected catheter and distal plug, but it collects only a small and spatially restricted amount of mucosal material and may therefore be affected by regional heterogeneity and low microbial biomass [234,235]. Thus, BALF provides broader but more diluted and potentially contamination-prone sampling, whereas PSB offers more localized and protected sampling. For both methods, procedural blanks and extraction controls are essential to identify reagent- and laboratory-derived contamination. In alternative the sputum offers a less invasive alternative, though with greater oropharyngeal contamination potential, while the lung tissue biopsies from transplantation or resection provide tissue-associated profiles but remain limited by availability [236]. To address contamination in low-biomass pulmonary microbiome studies, negative controls should be included throughout sample collection, DNA extraction, amplification and sequencing, together with positive mock-community controls to assess technical accuracy and batch effects [234,237,238]. Samples and controls should be processed in a randomized and balanced manner, while reagent lots and processing batches should be recorded, because contaminant profiles may vary between reagents and over time [239,240].

Once the samples have been isolated, the next step is the extraction of microbial DNA. This phase is delicate because the amount of microbial genetic material is often very small, leading to an underrepresentation of certain taxa. To maximize the microbial DNA recovery, the extraction protocols provide mechanical lysis and differential centrifugations [241].

After DNA extraction, the most common analytical approaches are 16S rRNA gene sequencing and shotgun metagenomics. The choice depends on the aim of study, the available resources, and the quality and amount of the starting material. 16S rRNA gene sequencing is the most widely used method for characterizing the bacterial component of the pulmonary microbiome. Validated in clinical cohorts of healthy volunteers and respiratory patients, it remains a cost-effective workhorse for profiling the lower respiratory tract microbiome, and also to compare microbial diversity across samples or clinical groups, although it generally achieves only genus-level taxonomic resolution, and it detects DNA regardless of whether the microorganisms are alive or dead [232,242].

Shotgun metagenomics provides species/strain-level resolution and functional profiling but requires >20 M reads to overcome >99% host DNA background in low-biomass airway samples [231,242]. This means that it can potentially detect bacteria, fungi, viruses, and functional genes at the same time, offering both taxonomic and functional information in human pulmonary diseases. It also usually provides higher taxonomic resolution than 16S sequencing, but it remains a more expensive solution, where deeper sequencing is required, and demands more advanced bioinformatic analysis.

Following sequencing, bioinformatic pipelines are used to assess alpha and beta diversity as well as taxonomic composition. Alpha diversity describes the richness and diversity of microorganisms within a single sample, whereas beta diversity evaluates differences between samples or groups. Taxonomic analysis identifies which microbial taxa are present and in what relative abundance. These analyses are valuable because they allow comparisons between disease states, treatment groups, or anatomical sites [231].

Quantification of microbial biomass by 16S rRNA gene qPCR, digital PCR or validated spike-in approaches can help determine whether sequencing signals exceed background contamination [237,243]. Finally, contaminant-identification algorithms should be combined with analyses performed before and after contaminant removal and with sensitivity analyses using alternative filtering thresholds. Concordant findings across these approaches provide greater confidence that the detected taxa represent genuine pulmonary signals rather than technical artefacts [240,244].

Despite significant methodological advances, critical gaps remain. Many studies provide cross-sectional snapshots rather than longitudinal assessments of microbiome dynamics [15,107]. Standardization of sampling protocols, DNA extraction methods, and bioinformatic pipelines across research laboratories remains incomplete, hindering meta-analyses and reproducibility [15,242,245]. Studies integrating multi-omics approaches, such as microbiomics with metatranscriptomics [246], functional predictions [247], and proposed metabolomics [248], with clinical outcomes illustrate the need for sophisticated analytical methods and large, well-phenotyped cohorts to fully realize their diagnostic and/or prognostic potential. Quantitative approaches that measure absolute bacterial loads alongside compositional profiling are needed to distinguish true microbiome changes from dilution effects [107,249].

To complement observational findings from clinical cohorts and establish direct functional links, understanding how lung microbiome influences local and systemic immunity requires experimental systems that recapitulate the complex architecture and cellular composition of human airways [231,250]. Human lung organoids, derived from induced pluripotent stem cells or primary airway epithelium, have emerged as powerful ex vivo models that maintain differentiated epithelial cell types, mucus production, and ciliary function [250,251]. These three-dimensional culture systems enable controlled bacterial exposures while monitoring epithelial barrier integrity, innate immune signaling, and antimicrobial peptide production [250,251]. Furthermore, biomimetic co-culture systems and microfluidic organ-on-a-chip models, incorporating immune cells, particularly macrophages, dendritic cells, and neutrophils, alongside airway epithelium permit investigation of multicellular immune responses to commensal and pathogenic bacteria [250,252]. Insights gained from human BALF cohorts and murine models demonstrate that lung microbiome composition shapes both local cytokine production and systemic immune tone; hence, such integrative approaches must be more widely adopted to validate mechanistic hypotheses generated from clinical associations in pulmonary diseases [24,253,254].

The metabolic activities of lung microbiome fundamentally shape the local chemical environment through microbial-derived metabolites, influencing immune function, epithelial physiology, and pathogen susceptibility in chronic respiratory diseases, such as severe IgE-mediated asthma and COPD [247,248]. Indeed, dysbiotic communities often exhibit altered metabolic profiles characterized by accumulation of host- and microbial-derived inflammatory mediators, such as neutrophil-derived peptides resulting from high proteolytic activity, and indole metabolites, alongside reduced production of anti-inflammatory metabolites (like SCFAs), and shifts in oxidative stress markers [248,255].

The landmark study by Narayana et al. provides compelling evidence that the gut–lung microbial interaction is a critical determinant of disease outcome in bronchiectasis [256]. By integrating sputum and stool microbiome analyses, the authors demonstrated that a microbial axis driven by lung *Pseudomonas* and gut *Bacteroides* is associated with worse clinical outcomes. Importantly, their computational modeling and in vivo murine experiments further suggest that this gut–lung microbial crosstalk is not merely a marker of disease severity but may represent a therapeutically targetable inter-compartmental axis. These findings support the concept that gut-targeted interventions could be exploited to therapeutically modulate the lung microenvironment, offering a rationale for targeting the gut–lung axis as an innovative strategy to improve respiratory disease outcomes. Microbiome-restorative approaches may be particularly valuable in settings of profound dysbiosis, such as severe COPD [107,249]. To reverse such dysbiosis and restore pulmonary homeostasis, therapeutic manipulation of the microbiome has been actively explored. While the concept of microbiome transplantation for respiratory diseases has long been considered highly experimental, recent clinical data from the phase 2 FTM-LUMINate trial demonstrate that overcoming ecological hurdles via oral-fecal microbiota transplantation is clinically feasible, achieving an 80% objective response rate in NSCLC patients [257]. Furthermore, preclinical studies exploring lung microbiome or gut microbiota manipulation have demonstrated feasibility in restoring microbiome diversity and barrier function in antibiotic-treated murine models, where the elimination or reintroduction of specific bacterial taxa directly modulates therapeutic outcomes [257,258].

In recent years, probiotics, prebiotics, and postbiotics have attracted growing interest as therapeutic tools in pulmonary medicine. Through the gut–lung axis, these compounds may modulate systemic immunity and indirectly influence the pulmonary microenvironment, promoting immune balance and dampening chronic inflammation [148]. While probiotics promote the colonization of beneficial bacteria, prebiotics stimulate their growth and metabolic activity, postbiotics, such as bioactive microbial metabolites including SCFAs, which exert immunomodulatory effects without the risks associated with live microorganisms [259]. Together, these targeted gut- and lung-directed strategies represent an innovative frontier in prevention and management of both acute and chronic lung diseases [148,259].

### 4.1. Probiotics

Probiotics have shown promising therapeutic potential for pulmonary diseases, although evidence remains preliminary and strain-specific.

In murine models of lung carcinoma, the administration of *Lactobacillus acidophilus* alongside cisplatin significantly enhanced treatment efficacy, reducing tumor size and improving survival [260]. Conversely, depletion of the microbiota via antibiotics impaired CD8^+^ T cell activation and the expression of pro-apoptotic genes, weakening the antitumor response. These findings suggest that maintaining a balanced gut microbiota can not only boost the effectiveness of chemotherapy but also positively modulate the immune system against lung cancer, highlighting the potential of probiotic-based strategies to improve oncological outcomes [260].

Similarly, Ponholzer et al. examined the role of pulmonary microbiome in lung transplantation, showing that microbial alterations contributed both to the development of chronic lung diseases leading to transplantation and to post-transplant outcomes, including chronic lung allograft dysfunction [261]. The presence of specific species, such as *P. aeruginosa*, and the overall bacterial load were found to influence graft survival and function. Following transplantation, the lung microbiome can undergo significant changes due to immunosuppressive therapy, infections, and environmental factors, suggesting that targeted modulation of microbiome could serve as an alternative strategy to improve post-transplant clinical outcomes [261].

To properly evaluate microbiome-directed strategies, preclinical finding, surrogate systemic biomarkers, and direct clinical respiratory outcomes must be clearly distinguished. At the preclinical level, intranasal administration of *Lactobacillus* strains in a murine model reduces mortality, bacterial lung burden, and tissue damage following *P. aeruginosa* infection while modulating local immune responses. [262]. The treatment also modulated the local immune response, leading to better control of inflammation and enhanced bacterial clearance. These results indicate that targeted modulation of the respiratory microbiome via intranasal probiotics may represent a promising preclinical preventive strategy against acute bacterial pneumonia, although robust clinical trials are required before establishing any translational efficacy in humans [262]. Probiotics have also been proposed as an alternative therapy for COPD, complementing primarily palliative treatments. In animal models, strains of *Bifidobacterium* modulated humoral and cellular immunity, while *E. coli* Nissle 1917 reduced pulmonary inflammation. *Lactobacillus plantarum* acted on Treg cells, macrophages, neutrophils, reducing cytokine release, such as IL-6 and TNF-α. Probiotics administered intranasally enhanced the immune defenses of the respiratory tract, and *Lactobacillus casei* promoted immune cell migration and reduced lung pathogen infections. Regarding human clinical data, daily intake of *L. casei* could also boost natural killer cell activity in smoking patients with COPD [263]. However, evidence regarding direct respiratory clinical endpoints in human disease remains non-conclusive. Coffey et al. conducted a meta-analysis evaluating the efficacy and safety of probiotics in improving health outcomes in children and adults with CF. In these studies, probiotics, primarily *Lactobacillus* spp., *Bifidobacterium* spp., *Saccharomyces* spp., and *Streptococcus* spp., were generally administered orally as single- or multi-strain formulations [264]. The authors reported that probiotic supplementation significantly reduced fecal calprotectin levels, a marker of intestinal inflammation, in both pediatric and adult patients. However, probiotics appeared to have little or no effects on pulmonary exacerbation rates and were associated with a small number of adverse events, including vomiting, diarrhea, and allergic reactions [264]. Overall, these findings highlight that positive responses in murine models or gastrointestinal surrogate biomarkers cannot be extrapolated to prove established pulmonary clinical efficacy in humans.

Another study by Spacova et al. investigated the preventive effects of intranasal administration of *L. rhamnosus GG* in a murine model of birch pollen-induced allergic asthma [265]. Mice received repeated intranasal doses of probiotic before allergen exposure. The treatment significantly reduced eosinophil infiltration in BAL fluid, decreased Th2 cytokines (IL-5, IL-13) in lung tissue, and lowered airway hyperreactivity. The probiotic also persisted in the nasal mucosa, indicating colonization and local protective effects. These results demonstrate a strain-specific preventive effect of *Lactobacillus rhamnosus GG* against allergic asthma in mice [265]. Furthermore, the inhalation of probiotics, particularly *Lactobacillus* strains, can significantly reduce lung metastases in experimental models and enhance the efficacy of chemotherapy, suggesting that probiotics, also administered via inhalation, could represent a promising complementary approach to enhance the efficacy of chemotherapy and counteract the spread of lung metastases, highlighting the crucial role of the pulmonary microbiome in modulating the antitumor immune response [258].

### 4.2. Prebiotics

Prebiotics, like probiotics, show promising indications for pulmonary diseases, but evidence remains primarily preclinical with limited clinical trial data. The available evidence consists mainly of animal studies, as reported by Bezemer et al., which demonstrated that a symbiotic mixture with *Bifidobacterium* and short-chain galacto-oligosaccharides, long-chain fructo-oligosaccharides, and low-viscosity pectin, orally administered to mice with LPS-induced pulmonary neutrophilia, improved lung function by 35% and reduced neutrophil recruitment by 40.7% [266].

Furthermore, specific prebiotic components demonstrate significant lung-protective effects in vivo: components such as pectin-derived acid oligosaccharides (pAOS), reduced mortality and bacterial load in mice with *P. aeruginosa* lung infection while modulating inflammatory responses via gut microbiota-derived butyrate. Prebiotics, such as FOS and GOS, helped to control airway inflammation markers in a mouse model of allergic asthma [267,268]. Notably, FOS and GOS supplementation have also been extended to CF patients as a strategy to optimize the gut microbiota composition [269]. Specifically, these prebiotics together with inulin have been selected for their ability to promote the growth of beneficial bacteria, including *Bifidobacterium* and *Lactobacillus*, and to increase the production of SCFAs with anti-inflammatory effects. The study aims to emphasize the connection among gut microbiota, lung function, and clinical outcomes suggesting potential indirect benefits on respiration and disease progression.

Similarly, Sutedja et al. investigated resveratrol as an adjuvant prebiotic therapy in pulmonary thromboembolism, modulating the gut microbiota-derived metabolite production of trimethylamine N-oxide, which is noted to be a direct contributor to accelerated thrombogenesis and thus an enhancer of pulmonary thromboembolism susceptibility [270]. Resveratrol, a natural polyphenol found in grapes and berries, may modulate the gut–lung axis and the generation of beneficial microbial metabolites to favor anti-inflammatory and immunomodulatory effects, which may support vascular health, attenuate pulmonary vascular remodeling, and reduce thrombus formation in the lungs. Overall, resveratrol has been proposed as a complementary therapy to improve outcomes in pulmonary embolism, although further clinical studies are needed [270].

### 4.3. Postbiotics

Postbiotics are bioactive metabolites and structural components derived from probiotic microorganisms that exert health benefits without requiring live bacteria. These include SCFAs, exopolysaccharides, bacteriocins, and cell-free supernatants (CFS), bacterial cell wall fragments, and probiotic-derived EVs. In a respiratory condition context, such substances demonstrate benefits across multiple respiratory conditions, they exert anti-inflammatory, immunomodulatory, and antimicrobial effects. Specifically, postbiotics modulate immune responses by reducing pro-inflammatory cytokines, enhancing macrophage and NK cell activity, and influencing airway pathogens [271].

Pompilio et al. investigated the activity of CFS obtained from *Lactobacillus* strains against *P. aeruginosa* isolated from CF patients, presenting a promising postbiotic approach along the gut–lung axis to counteract chronic respiratory infections in CF [272]. The authors have evaluated the antibacterial, antibiofilm, and antivirulence effects of several probiotic-derived supernatants in vitro (under mimicking the CF lung) and in vivo in a *Galleria mellonella* model, showing that these postbiotics were able to inhibit the growth of *P. aeruginosa* particularly at acidic pH, affecting the production of virulence factors, such as elastase and pyocyanin, and biofilm formation without showing host toxicity. The observed activity was attributed to bioactive molecules produced by *lactobacilli*, including organic acids and other antimicrobial compounds, such as diketopiperazine 3-isobutyl 2,5 piperazinedione, demonstrating how gut-derived metabolites can directly target pulmonary pathogens [272].

The potential use of postbiotics as an adjuvant strategy in the treatment of SARS-CoV-2 infection has been reported [273]. Due to their high stability within the gastrointestinal tract and favorable safety profile, postbiotics may also exert indirect antiviral effect along the gut–lung axis. Specifically, they modulate angiotensin-converting enzyme 2 activity, contributing to maintaining intestinal barrier integrity and microbiota balance.

However, a significant limitation of current research on postbiotics is the lack of quantified clinical data, such as robust sample sizes and clear effect sizes in studies on pulmonary applications, as the available evidence remains predominantly preclinical.

Probiotics, prebiotics, and postbiotics have been studied for their potential in managing pediatric asthma [274]. Probiotics, mainly including *L. rhamnosus*, *Lactobacillus casei*, *Lactobacillus acidophilus*, *Bifidobacterium breve*, and *Bifidobacterium longum*, administered as oral single- or multi-strain formulations, demonstrated reductions in asthma exacerbation. Postbiotics, specifically bacterial lysates, such as OM-85 BV and PMBL^®^, have been shown to reduce airway inflammation and hyperreactivity. Prebiotics, including FOS and GOS, evaluated primarily within symbiotic formulation, support beneficial gut bacteria, modulating immune responses, and indirectly influencing lung health via the gut–lung axis. These interventions appear to regulate Th1/Th2 cytokine release, suppressing Th2-related IL-4, IL-5 and IL-13 release while enhancing Th1-related IFN-γ, and induce regulatory T cells secretion of anti-inflammatory IL-10 and TGF-β. These changes improved lung function and reduced the frequency and severity of acute respiratory exacerbations, including asthma attacks and bronchospasm episodes, although variability in strains, dosages, and treatment duration highlights the need for further well-designed clinical studies [274].

Despite promising results, clinical evidence on the efficacy of probiotics, prebiotics, and postbiotics in respiratory diseases remains preliminary. Major limitations include the heterogeneity of bacterial strains or metabolites used, the doses administered, routes of delivery, and study endpoints. The wide variability of experimental protocols makes it difficult to draw definitive conclusions on therapeutic effectiveness and standardization of clinical treatments. Therefore, larger, well-designed, and controlled clinical trials are needed to precisely define the role of probiotics, prebiotics, and postbiotics as complementary tools in the management of respiratory diseases.

## 5. Conclusions

Collectively, current evidence identifies mitochondria as important regulators of the inflammatory, metabolic, immune and microbial responses in lung diseases. The pulmonary microbiome, once considered a negligible component of respiratory biology, is now recognized as a dynamic and low-biomass ecosystem associate with immune homeostasis, epithelial barrier function, and susceptibility to infection. However, a universal healthy lung microbiome cannot be defined because the microbial profiles detected in respiratory samples depend substantially on anatomical location, specimen type, upper-airway carryover, microbial load, DNA extraction procedures, sequencing platforms, bioinformatic pipelines, and contaminant-removal strategies. Genera such as *Streptococcus*, *Veillonella*, *Prevotella*, *Haemophilus*, *Neisseria*, *Corynebacterium*, and *Propionibacterium* have frequently been detected in healthy respiratory samples, but their relative abundance and biological relevance vary across studies. Although resident airway microorganisms may contribute to ecological stability and immune regulation, direct evidence for a generalized colonization-resistance mechanism in the human lung remains limited. Conversely, disruption of pulmonary microbial composition, diversity, or bacterial burden has been associated with chronic inflammation, impaired pathogen clearance, and disease progression across CF, COPD, ARDS, IPF, asthma, bronchiectasis, and lung cancer. Most of these clinical observations remain associative and may also be influenced by disease severity, antibiotic exposure, sampling procedures, and other confounding factors.

A key conclusion emerging from this review is that pulmonary dysbiosis and mitochondrial dysfunction can intersect in the regulation of pulmonary inflammation, although the evidence supporting the two directions of this interaction is not equally strong. Dysbiotic microbial communities and respiratory pathogens can directly induce mitochondrial stress in epithelial and immune cells through bacterial toxins, virulence factors, microbial metabolites, and pattern-recognition receptor activation. These stimuli promote mitochondrial ROS production, mitochondrial Ca^2+^ overload, loss of membrane potential, impaired oxidative phosphorylation, defective mitophagy, and release of mtDAMPs, including oxidized mtDNA and mitochondrial-derived vesicles. These findings underscore that selected microbial factors may alter mitochondrial function and thereby amplify host inflammatory responses. In turn, mitochondrial dysfunction may influence epithelial repair, redox balance, immunometabolism, nutrient availability, mucociliary clearance, and antimicrobial responses, which potentially modifying microbial composition and or diversity. However, direct evidence that mitochondrial dysfunction determines the overall architecture of the human pulmonary microbiome remains limited. The proposed bidirectional relationship should therefore be interpreted as a context-dependent model in which some individual mechanisms are experimentally demonstrated.

The consequences of these interactions for airway remodeling and mucus secretion are also likely to be indirect and disease specific. In CF, defective CFTR-dependent ion and fluid transport remains the primary cause of mucus dehydration and impaired mucociliary clearance. Microbial infection, Ca^2+^ and mitochondrial dysregulation, oxidative stress, inflammasome activation, and neutrophilic inflammation may subsequently amplify epithelial injury and mucus obstruction. In COPD, mitochondrial dysfunction may contribute to oxidative stress, cellular senescence, impaired epithelial restitution, and altered responses to microorganisms, but airway remodeling also depends on cigarette-smoke, chronic inflammation, structural injury, and recurrent infections. Mitochondrial and microbial abnormalities should therefore be considered interacting contributors to these complex pathological processes.

These mechanisms have important clinical implications. Microbiome composition, bacterial load, microbial metabolites, and host mitochondrial stress markers could potentially provide complementary information for disease stratification, prognosis, and therapeutic monitoring. In diseases such as CF and COPD, reduced microbial diversity and dominance of pathogenic taxa correlate with inflammation and clinical decline, while in ARDS and IPF, microbial signatures may help identify patients at higher risk of severe outcomes. Moreover, the gut–lung axis adds an additional level of clinical relevance. Experimental studies indicate that intestinal microorganisms, microbial products, and metabolites can influence systemic immunity and pulmonary inflammatory responses. Gut-barrier disruption and microbial translocation may contribute to systemic and pulmonary inflammation during critical illness, including ARDS, but the directionality and clinical magnitude of these interactions remain difficult to establish in patients.

Therapeutically, strategies aimed at restoring microbiome balance and preserving mitochondrial function have generated encouraging findings in experimental systems. Probiotics, prebiotics, postbiotics, SCFAs, microbiota-restorative interventions, mitochondrial antioxidants, modulators of mitochondrial Ca^2+^ signaling, and EV-based therapies have all shown potential in experimental settings. However, clinical translation remains limited by heterogeneity in study design, strain specificity, dosing, delivery route, patient selection, and endpoints.

Future research should therefore prioritize longitudinal, multi-center studies integrating microbiomics, metatranscriptomics, metabolomics, host transcriptomics, immunophenotyping, and mitochondrial functional analyses. Attention should be paid to temporal relationships, because cross-sectional studies cannot determine whether microbial alterations precede mitochondrial dysfunction, develop as a consequence of tissue injury, or reflect disease severity and treatment exposure. Mechanistic investigations should directly manipulate specific microorganisms, microbial products, host metabolites, and mitochondrial pathways to establish which components are necessary and sufficient for the observed biological effects. Standardized sampling protocols, contamination controls, DNA extraction methods, quantitative bacterial load measurements, and harmonized bioinformatic pipelines are essential to improve reproducibility. Advanced models, including human lung organoids, lung-on-chip platforms, and epithelial–immune co-culture systems, should be more widely adopted to validate mechanistic hypotheses derived from clinical associations. Ultimately, a system-level understanding of mitochondria–microbiome crosstalk may contribute to the development of more precise diagnostic and therapeutic approaches. Nevertheless, the mitochondria–microbiome relationship should not yet be interpreted as a uniformly demonstrated bidirectional axis across all respiratory disorders. Current evidence supports direct effects of selected pathogens and microbial factors on mitochondrial function than a generalized capacity of mitochondrial dysfunction to reshape pulmonary microbial-community architecture. Distinguishing experimentally demonstrated mechanisms, context-dependent hypotheses, and clinical associations will be essential before microbial, metabolic, immune, and mitochondrial biomarkers can be reliably integrated into personalized management strategies for inflammatory and infectious lung diseases.

## Data Availability

No new data were created or analyzed in this study. Data sharing is not applicable to this article.

## List of Abbreviations

ARDS	Acute respiratory distress syndrome
ARF	Acute Respiratory Failure
ATP	Adenosine Triphosphate
ASC	Apoptosis-associated Speck-like protein containing a CARD
GABA	4-aminobutyrate
BALF	Bronchoalveolar lavage fluid
CEA	Carcinoembryonic antigen
CAMP	Cathelicidin Antimicrobial Peptide
CFS	Cell-free supernatants
CXCL1	Chemokine C-X-C ligand 1
COPD	Chronic obstructive pulmonary disease
CF	Cystic fibrosis
CFTR	Cystic fibrosis transmembrane conductance regulator
CYFRA21-1	Cytokeratin 19 fragments
DNA	Deoxyribonucleic acid
DEFA4	Defensin Alpha 4
ERK1/2	Extracellular signal-regulated kinases 1 and 2
EVs	Extracellular Vesicles
FOS	Fructo-oligosaccharides
GOS	Galacto-oligosaccharides
GM-CSF	Granulocyte-Macrophage Colony-stimulating Factor
HAP	Hospital-Acquired Pneumonia
H_2_S	Hydrogen sulfide
IPF	Idiopathic pulmonary fibrosis
IL-1RA	IL-1 Receptor Antagonist
IgA	Immunoglobulin A
iNOS	Inducible nitric oxide synthase
IFN-γ	Interferon-gamma
IL-1β	Interleukin-1β
LC3	Light Chain 3
LPS	Lipopolysaccharide
Treg	Lymphocyte regulatory T cells
Th1	Lymphocyte T helper 1
MIP-1β/CCL4	Macrophage inflammatory protein-1β
MMP9	Matrix metalloproteinase-9
MCU	Mitochondrial calcium uniporter
mtDAMPs	Mitochondrial damage-associated molecular patterns
mtDNA	Mitochondrial DNA
mPTP	Mitochondrial permeability transition pore
mtROS	Mitochondrial ROS
MitoVit-E	Mitochondrial vitamin E
MAPK	Mitogen-Activated Protein Kinase
Mito-Q	Mitoquinone
NK	Natural killer
NSE	Neuron-specific Enolase
NET	Neutrophil extracellular trap
NADH	Nicotinamide Adenine Dinucleotide
NADPH	Nicotinamide Adenine Dinucleotide Phosphate
NLRC4	NLR family CARD Domain Containing 4
NLR	NOD-like receptor
NLRP3	NLR family pyrin domain-containing 3
NSCLC	Non-small cell lung cancer
NDP52	Nuclear Domain 10 Protein 52
NF-κB	Nuclear Factor Kappa-light-chain-enhancer of activated B cells
pAOS	Pectin-derived acid oligosaccharides
PGLYRP1	Peptidoglycan Recognition Protein 1
PGC-1α	Peroxisome proliferator-activated receptor gamma coactivator 1-alpha
PTEN	phosphatase and tensin homolog
PCR	Polymerase Chain Reaction
pIgR	Polymeric immunoglobulin receptor
PSB	Protected specimen brush
PINK1	PTEN-induced kinase 1
RNS	Reactive nitrogen species
ROS	Reactive oxygen species
RIG-I	Retinoic acid-inducible gene I
SLPI	Scecretory Leukocyte Protease Inhibitor
SCFAs	Short-chain fatty acids
T3SS	Type 3 Secretion System
TLR-4	Toll-Like Receptor 4
TFAM	Transcription Factor A mitochondrial
TGF-β	Transforming Growth Factor-beta
TNF-α	Tumor Necrosis Factor-alpha
